# Targeting Sphingosine‐1‐Phosphate Signaling Attenuates Doxorubicin‐Aggravated Bone Loss in Obese Breast Cancer Mice

**DOI:** 10.1002/smmd.70031

**Published:** 2026-03-30

**Authors:** Yu Zhang, Hao Shen, Junjie Niu, Yingkang Huang, Can Zhu, Yi Wang, Yida Chen, Xinyi Cheng, Huilin Yang, Xianrong Zhang, Hao Chen, Hongbo Zhang, Qin Shi

**Affiliations:** ^1^ Department of Orthopedics The First Affiliated Hospital of Soochow University Orthopedic Institute of Soochow University Suzhou Jiangsu China; ^2^ Pharmaceutical Sciences Laboratory Faculty of Science and Engineering Åbo Akademi University Turku Finland; ^3^ Department of Orthopedics Nanfang Hospital Southern Medical University Guangzhou Guangdong China; ^4^ Medical College Yangzhou University Yangzhou Jiangsu China; ^5^ Turku Bioscience Centre University of Turku and Åbo Akademi University Turku Finland

**Keywords:** bone loss, breast cancer, doxorubicin, obesity, sphingosine‐1‐phosphate

## Abstract

Although chemotherapy‐induced bone loss is well‐recognized during breast cancer treatment, the underlying mechanism remains to be further elucidated, especially in patients with obesity. In this study, the objective was to investigate the impact of genomic silencing and pharmacological inhibition of S1P synthesis on bone loss in doxorubicin‐induced obese breast cancer mice. In vitro study, upon the treatment of doxorubicin combined with palmitic acid, the S1P generated by 4T1 cells was significantly increased, resulting in an increase in osteoclastogenesis by activating the S1PR1/p‐STAT3/NFATc‐1 pathway in bone marrow‐derived macrophages. In vivo study, pharmacological intervention with Sphingosine kinases (SPHK) antagonist SKI II or biological inhibition with SPHK1 and SPHK2 short hairpin RNA significantly reduced S1P production and rescued the obese breast cancer‐bearing mice from doxorubicin‐induced bone loss, manifested by the decreased osteoclastogenesis and recovered bone microarchitecture. Similarly, the administration of the S1PR1 antagonist FTY720 also alleviated bone loss in the breast cancer‐bearing mice fed a high‐fat diet. These studies indicate that genetic silencing and pharmacological inhibition can suppress S1P‐dependent bone loss in doxorubicin‐induced obese breast cancer mice. S1P shows promise as a potential drug target for preventing chemotherapy‐induced bone loss in patients.

## Summary

- DOX increases S1P secretion from breast cancer cells upon fatty acid intervention and induces osteoclastogenesis through the activation of the S1PR1/p‐STAT3/NFATc‐1 pathway.
- Inhibition of S1P generation rescues cancer chemotherapy‐induced bone loss in breast cancer‐bearing mice fed a high‐fat diet.

## Graphical abstract

During obese breast cancer‐bearing mice undergoing chemotherapy, DOX increases the concentration of S1P produced by breast cancer cells, which induces osteoclastogenesis through the activation of the S1PR1/p‐STAT3/NFATc‐1 pathway, thereby exacerbating bone loss in the breast cancer‐bearing mice with high‐fat dietary.

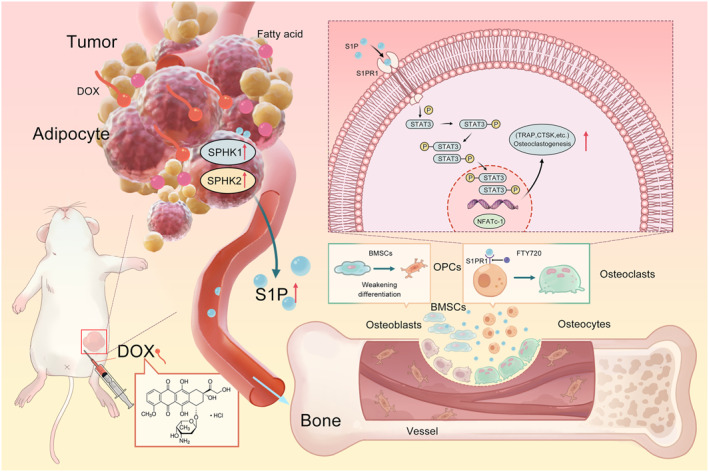

## 1 Introduction

Since 2021, breast cancer has surpassed lung cancer to become the most prevalent type of cancer globally [1]. With a propensity of breast cancer for metastasizing to the skeleton, up to 85% of patients will harbor bone metastases, posing severe threats to the patient's prognosis [2, 3]. Cancer cells disrupt bone homeostasis and structural integrity, increasing the risk for skeletal‐related events such as bone loss and bone fracture. Substantial epidemiological evidence has confirmed that obesity increases the incidence and mortality of cancers due to the metabolic symbiosis between adipocytes and cancer cells [4, 5]. Obese patients with breast cancer, relative to normal‐weight ones, bear an additional 35% of the risk for cancer‐specific mortality [6]. Of note, an increased fracture risk in obese breast cancer‐bearing patients was also revealed in recent studies, but the underlying mechanism remains unclear [7]. With increasing morbidities in obesity, the complicated relationship between breast cancer and obesity poses unique diagnostic and therapeutic challenges to clinicians.

Among all the treatment options for breast cancer, chemotherapy has been established as a highly effective treatment for breast cancer at every stage, with remarkable advantages in slowing the proliferation of malignant cells and extending patient survival rates. However, chemotherapy can negatively impact bone health, especially in women with breast cancer [8]. Surprising evidence presented a notable decline in bone mineral density (BMD) at the hip and lumbar spine among premenopausal breast cancer patients undergoing chemotherapy [9]. For breast cancer patients with obesity, more complications and less effectiveness related to chemotherapy were revealed [7]. Chemotherapeutic drugs such as cyclophosphamide, methotrexate, and fluorouracil can induce rapid bone loss in postmenopausal breast cancer patients [10]. As a typical example of chemotherapeutic drugs, doxorubicin (DOX) is also known to induce bone loss. Although it has been widely used in cancer treatment for over 40 years, the mechanism behind DOX‐mediated cancer‐related bone loss, especially in breast cancer patients with obesity, is poorly defined.

Sphingosine‐1‐phosphate (S1P) is a bioactive sphingolipid generated through the phosphorylation of sphingosine, a reaction catalyzed by sphingosine kinase 1 (SPHK1) and sphingosine kinase 2 (SPHK2). This molecule is involved in numerous biochemical processes, including cell proliferation, differentiation, migration, and the production of cytokines and chemokines [11, 12]. Recent studies have shown that S1P is crucial in diverse pathological conditions such as cancer and bone diseases [13, 14, 15, 16]. On the cellular level, S1P can enhance growth, proliferation, angiogenesis, metastasis, and cancer cell survival via interaction with its cell surface receptor S1PR1 [17]. Clinical evidence revealed that the blood concentration of S1P was significantly elevated in obese breast cancer patients, indicating a potential function of S1P in cancer development [18]. In bone remodeling, S1P was also considered as a regulator of osteoclast‐osteoblast coupling. Increased SPHK1 expression and activity were observed during osteoclast differentiation, resulting in high S1P levels [13, 19]. Due to the dual effects of S1P on cancer development and bone remodeling, we speculate that S1P participates in chemotherapy‐induced osteoporosis, especially in obese cancer patients.

**SCHEME 1:**
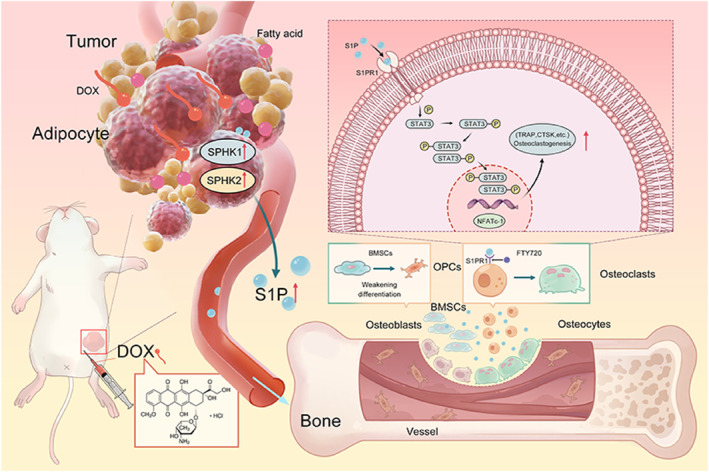
During obese breast cancer‐bearing mice undergoing chemotherapy, DOX increases the concentration of S1P produced by breast cancer cells, which induces osteoclastogenesis through the activation of the S1PR1/p‐STAT3/NFATc‐1 pathway, thereby exacerbating bone loss in the breast cancer‐bearing mice with a high‐fat diet.

In this study, we uncovered an S1P‐mediated crosstalk between the tumor microenvironment and bone microenvironment under obesity conditions. Specifically, S1P was first identified to be enhanced in the obese breast cancer‐bearing mice, resulting in an increase in osteoclastogenesis by activating the S1PR1/p‐STAT3/NFATc‐1 pathway of bone marrow‐derived macrophages. As expectedly, by inhibiting the S1P production with the pharmacological administration of SPHK antagonist SKI II or SPHK1/SPHK2 shRNA pharmacologically, the side effect of bone loss caused by DOX treatment was significantly alleviated in the obese breast cancer‐bearing mice (Scheme 1). The findings provide specific theoretical support and highlight potential therapeutic targets for bone loss caused by chemotherapy in the obese cancer patients.

## 2 Results

### 2.1 DOX Exacerbates Bone Loss in the Obese Mice Bearing Breast Cancer

**FIGURE 1:**
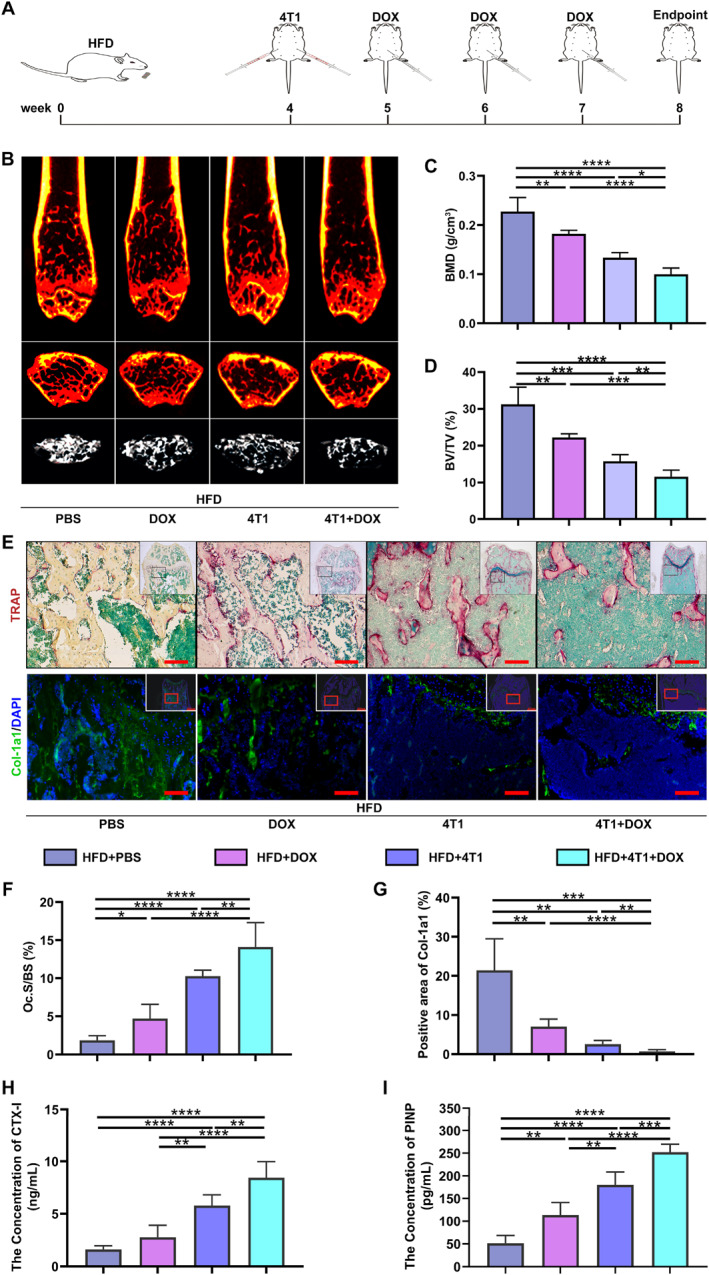
DOX exacerbates bone loss in the obese mice bearing breast cancer. (A) The schematic diagram of the in vivo experimental protocol. (B) The representative μ‐CT images of the femurs. Upper: coronal sections; Middle: cross sections; Lower: three‐dimensional images. (C) BMD and (D) BV/TV of the mice. (E) The representative TRAP staining images (upper) and Col‐1a1 staining images (lower). (F) The ratio of osteoclast surface area to bone surface area. (G) Quantification of Col‐1a1 positive staining. (H) and (I) Blood concentrations of CTX‐I and PINP. *n* = 4. Scale bar: 100 μm. *p < 0.05, **p < 0.01, ***p < 0.001, and ****p < 0.0001.

To investigate how cancer chemotherapy influences bone loss under obesity conditions, we established a breast cancer orthotopic mouse model concurrent with HFD and normal diet, and 4 weeks later, applied DOX treatment (Figure 1A). After 8 weeks, the body weight of the three groups (HFD+4T1, HFD+DOX, and HFD+4T1+DOX) was notably decreased than that of the control group (HFD+PBS, Supporting Information S1: Figure S1A). The tumor volume of the HFD+4T1+DOX group was markedly reduced from that of the HFD+4T1 group, indicating the effectiveness of DOX chemotherapy (Supporting Information S1: Figure S1B). Representative H&E staining and μ‐CT images of the distal femurs of all the groups revealed significant bone loss induced by breast cancer, which was aggravated after DOX treatment (Figure 1B and Supporting Information S1: Figure S1C and Figure S2). Specifically, DOX reduced the BMD and BV/TV values by 14.58% and 13.42% in the tumor‐bearing groups (HFD+4T1+DOX vs. HFD+4T1) (Figure 1C,D). Similar findings were indicated regarding the parameters such as BS/BV, BS/TV, Tb. N, and Tb. Sp (Supporting Information S1: Figure S1D‐G). Furthermore, compared with tumor‐bearing mice fed with a normal diet, tumor‐bearing mice receiving DOX treatment and maintained on a high‐fat diet exhibited more rapid and severe bone loss (Figure 1B–D and Supporting Information S1: Figure S2).

Bone turnover, a coupled process of bone resorption and formation, was assessed by the histological staining. Osteoclasts and osteoblasts were visualized by TRAP and Col‐1a1 immunofluorescent staining, respectively (Figure 1E). Quantitative analysis demonstrated a marked increase in osteoclast numbers and a substantial reduction in the positive area of Col‐1a1 in the HFD+4T1+DOX group (Figure 1F,G). ELISA analysis for CTX‐I and PINP was also performed in all mice. As a result, 4T1 cell injection and DOX chemotherapy increased the serum concentrations of CTX‐I and PINP (Figure 1H,I). In particular, compared to the HFD+4T1 group, the levels of CTX‐I and PINP in the HFD+4T1+DOX group were statistically upregulated by 1.42‐fold and 1.43‐fold, suggesting an increased rate of bone turnover provoked by DOX. These results indicate that DOX chenotherapy aggravates bone loss induced by breast cancer in obese mice.

### 2.2 DOX Increases the Production of S1P in the Breast Cancer Cells Under Obese Conditions

**FIGURE 2:**
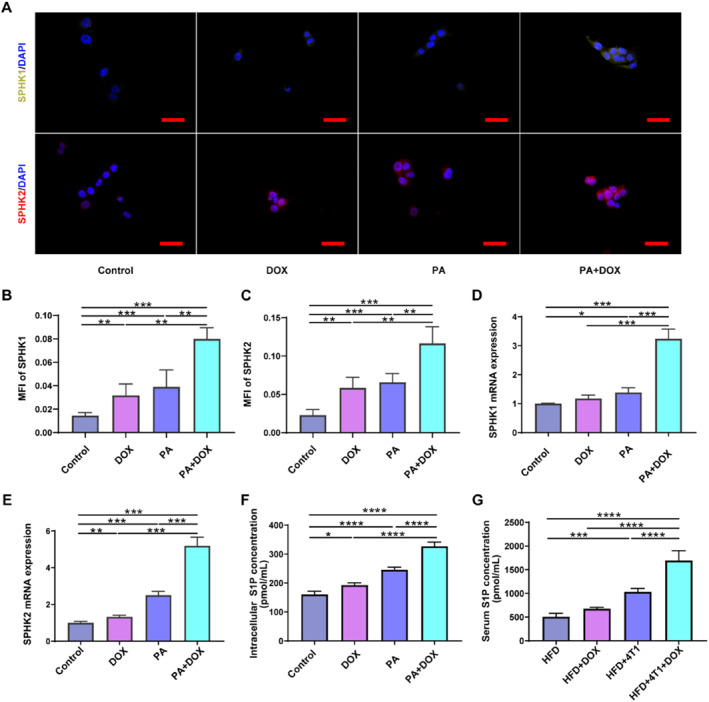
DOX increases the production of S1P in breast cancer cells upon obese condition. (A) Immunofluorescence staining for SPHK1and SPHK2 of 4T1 cells. (B) and (C) The mean fluorescence intensity (MFI) of SPHK1 and SPHK2. (D) and (E) Relative gene expression of Sphk1 and Sphk2. (F) and (G) The S1P concentrations in tumor cells and animal serum. (*n* = 4). Scale bar: 50 μm. *p < 0.05, **p < 0.01, ***p < 0.001, and ****p < 0.0001.

S1P has emerged as a vital regulatory molecule in breast cancer and bone‐related diseases. To probe the role of S1P in breast cancer chemotherapy‐induced bone loss in obese mice, we first examined the production of S1P in the 4T1 cells treated with PA and DOX. The expressions of SPHK1 and SPHK2, ubiquitous enzymes that generate S1P, were also detected by immunofluorescence (IF) staining (Figure 2A). Quantitative analysis showed significantly increased fluorescence intensities of SPHK1 and SPHK2 were observed in the PA+DOX group (Figure 2B,C), compared with the other three groups, control, PA, and DOX groups. In line with the results of IF staining, relative mRNA expressions of Sphk1 and Sphk2 were markedly elevated in the PA+DOX group (Figure 2D,E). Thus, DOX combined with PA significantly increased the production of S1P in 4T1 cells (Figure 2F).

Similar to the in vitro results, the serum concentration of S1P in the HFD+4T1+DOX group was also elevated compared to the other three groups (HFD+PBS, HFD+4T1, and HFD+DOX group, Figure 2G). These results indicate that DOX increases the production of S1P in the breast cancer cells under obese conditions.

### 2.3 DOX‐Treated 4T1 Cells in the Presence of PA Promote BMM Osteoclastogenesis Through S1PR1/P‐STAT3/NFATc‐1 Signaling Pathway

**FIGURE 3:**
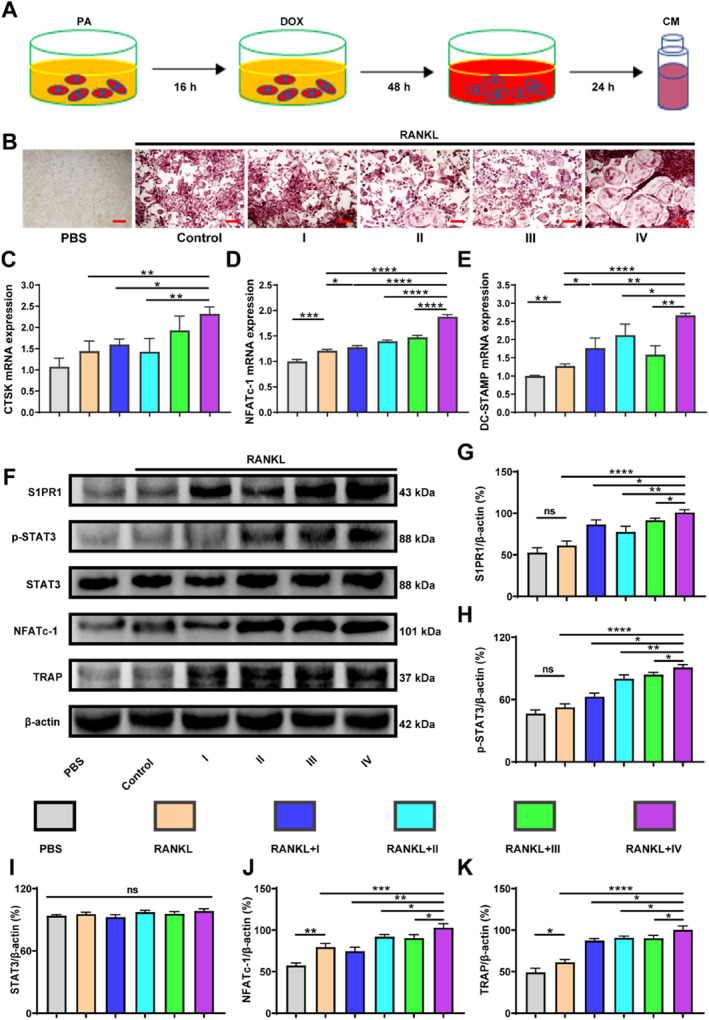
DOX combined with PA impairs bone homeostasis by promoting osteoclastogenesis in vitro. (A) The schematic illustration of the in vitro experimental protocol. (B) The representative images of TRAP staining of BMM‐derived osteoclasts. (C–E) Relative gene expression of *Ctsk*, *Nfatc‐1 and Dc‐stamp*. (F) WB of S1PR1, p‐STAT3, STAT3, NFATc‐1, TRAP, and β‐actin. (G–K) Relative protein expression of S1PR1, p‐STAT3, STAT3, NFATc‐1, TRAP. I: 4T1+PBS; II: 4T1+DOX; III: 4T1+PA; IV: 4T1+PA+DOX. *n* = 3, Scale bar: 200 μm. *p < 0.05, **p < 0.01, ***p < 0.001, and ****p < 0.0001.

To investigate how DOX influences osteoclasts in vitro, we applied PA into the culture medium of 4T1 cells to simulate the in vivo microenvironment of obese mice bearing breast cancer, and then administered DOX (Figure 3A). The optimal concentrations of PA and DOX for cell viability were determined to be 20 μM and 50 nM by the Cell Counting Kit‐8 assay (CCK‐8, Supporting Information S1: Figure S3A). After getting rid of PA and DOX in the cell culture of 4T1 cells for 24 h, the cell medium was collected as CM to induce BMM osteoclastogenesis, and the ideal ratio of CM to α‐MEM for osteoclast differentiation was found to be 1:9 (Supporting Information S1: Figure S3B‐E).

Seven days after osteoclastic induction, BMMs were differentiated into the most osteoclasts in the group pretreated with PA and DOX (Group IV), compared with the other three groups (Group I, II, and III; Figure 3B). The expression of osteoclast‐related genes, including cathepsin K (Ctsk), nuclear factor of activated T cells 1 (Nfatc‐1), dendritic cell‐specific transmembrane protein (Dc‐stamp), Sphingosine 1‐phosphate receptor 1 (S1pr1), and Signal transducer and activator of transcription 3 (Stat3), was statistically upregulated in Group IV than in the other groups (Figure 3C–E and Supporting Information S1: Figure S4A,B).

To investigate the mechanism of osteoclast formation, the receptor S1PR1 of S1P and its downstream signaling were examined. Flow cytometry and Western blot analyses were conducted after 24 h or 3 days of osteoclastic induction. The proportion of CD11b+S1PR1+ cells significantly increased in the BMM‐derived osteoclasts, especially in Group IV (Supporting Information S1: Figure S4C‐D). Additionally, the protein expression of S1PR1, p‐STAT3, STAT3, NFATc‐1, and TRAP in Groups I–IV was notably higher than that in the control and RANKL groups. Furthermore, the expression of these proteins in Group IV was notably higher than that in the other groups (Figure 3F–K). These results indicate that the combined stimulation of 4T1 cells by DOX and PA may promote BMM osteoclastogenesis through the S1PR1/p‐STAT3/NFATc‐1 signaling pathway.

Osteoblast differentiation is another vital process in bone homeostasis. To examine the effects of DOX‐treated tumor cells on osteogenesis, we induced MC3T3‐E1 cells into osteoblasts with different CMs and performed ALP staining and Alizarin Red S staining to detect the osteogenesis of MC3T3‐E1 cells. ALP activity and overall mineralization were significantly reduced in the groups treated with different 4T1 cell‐derived CMs (Groups I to IV) compared to the group treated with osteogenic medium only (the OB group). However, there was no notable difference observed between the four groups that were treated with the different CMs (Supporting Information S1: Figure S5A–D). These results indicate that both DOX and PA exacerbate cancer‐induced bone loss mainly by promoting osteoclastogenesis rather than inhibiting osteoblastogenesis.

### 2.4 FTY720 Antagonizes S1PR1‐Induced Osteoclastogenesis

**FIGURE 4:**
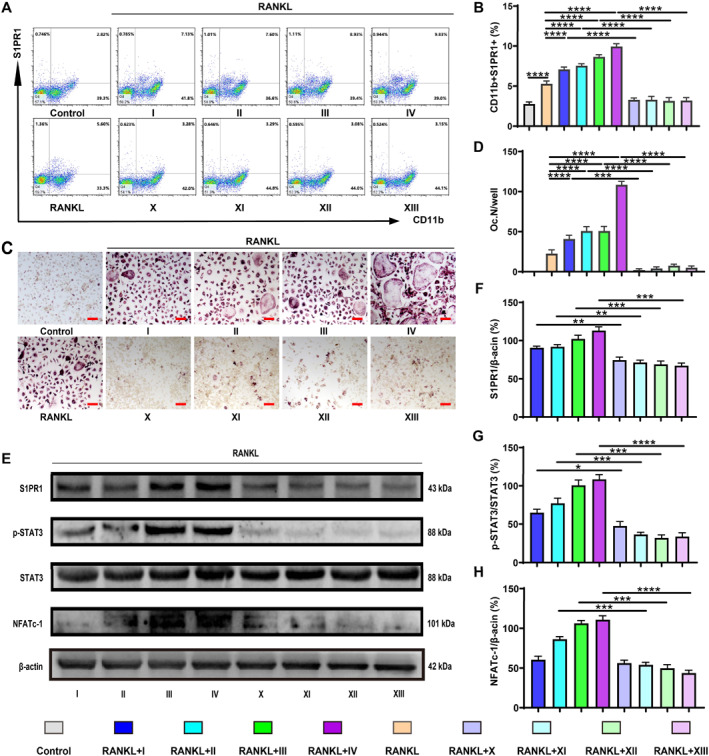
FTY720 antagonizes S1P‐induced osteoclastogenesis (A) Analysis of CD11b+S1PR1+ cells of BMMs by flow cytometry. (B) The proportion of CD11b+S1PR1+ cells. (C) Representative images of TRAP staining of BMM‐derived osteoclasts. (D) Quantification of mature osteoclasts. (E) WB analysis of S1PR1, p‐STAT3, STAT3, NFATc‐1, and β‐actin. (F and H) Quantification of S1PR1, NFATc‐1 relative to β‐actin. (G) Quantification of p‐STAT3 relative to STAT3. The CMs were I: 4T1+PBS; II: 4T1+DOX; III: 4T1+PA; IV: 4T1+PA+DOX; X: I+FTY720; XI: II+FTY720, XII: III+FTY720, XIII: IV+FTY720. *n* = 3. Scale bar: 200 μm. Mean ± SD is shown. *p < 0.05, **p < 0.01, ***p < 0.001, and ****p < 0.0001.

FTY720 has been approved by the FDA as an immunosuppressive agent and can induce a reduction in S1PR1 expression in macrophages within tissues [20, 21, 22]. We subsequently investigated the impact of FTY720 on S1PR1 expression in BMM‐derived osteoclasts during the osteoclastic induction with CMs for 3 days. Corresponding to the trend of WB, FCA showed that S1PR1 expression increased in all the groups of BMM‐derived osteoclasts (CD11b+S1PR1+) during osteoclastogenesis with CM addition (Figure 4A,B). Compared with the other three groups with CM addition, S1PR1 expression was enhanced mostly in the 4T1+PA+DOX group. However, S1PR1 expression was significantly inhibited following the delivery of FTY720, whereas no significant differences were observed among all groups with CM addition. WB results displayed the same outcome (Figure 4E,F), which suggested that FTY720 successfully inhibited the expression of S1PR1 in BMM‐derived osteoclasts.

Remarkably, when FTY720 induced a reduction in S1PR1 expression in BMMs, the expression of p‐STAT3 and NFATc‐1 was also significantly decreased (Figure 4D,G,H). Furthermore, along with the reduction in S1PR1/p‐STAT3/NFATc‐1 expression, the generation of TRAP‐positive cells in BMM‐derived osteoclasts after 7 days of induction was significantly suppressed (Figure 4C,D), compared with the groups without FTY720 administration. In other words, FTY720 reduces the expression of S1PR1 in BMM‐derived osteoclasts, thereby blocking S1P‐induced osteoclastogenesis through the S1PR1/p‐STAT3/NFATc‐1 signaling pathway.

### 2.5 SKI II Inhibits S1P‐Induced Osteoclastogenesis

**FIGURE 5:**
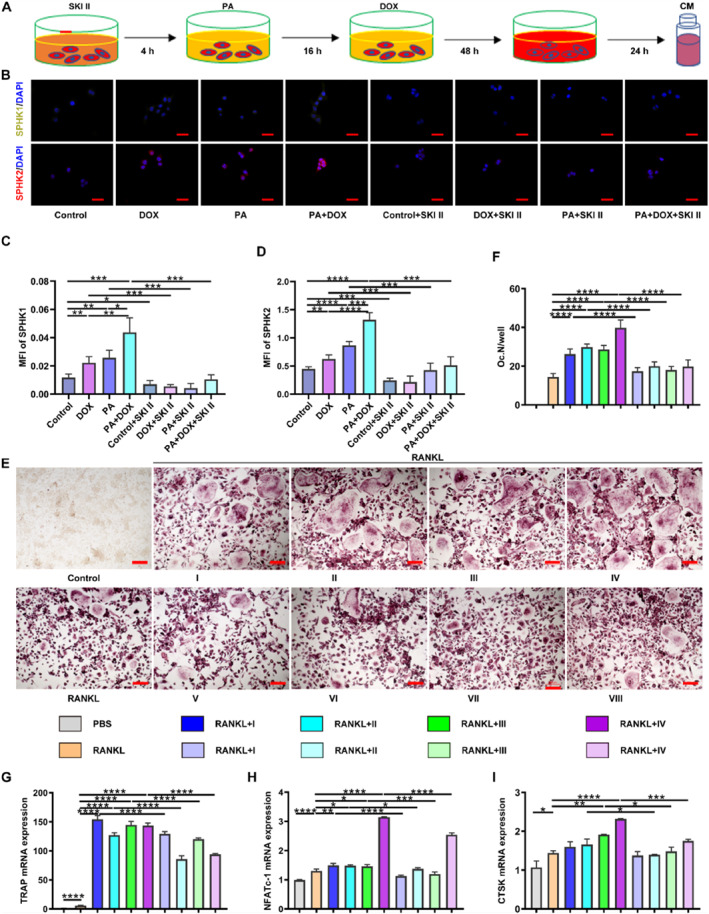
SKI II inhibits S1P‐induced osteoclastogenesis. (A) Schematic diagram of in vitro experimental protocol. (B) Immunofluorescence staining for SPHK1 and SPHK2 of 4T1 cells. Scale bar: 50 μm. (C, D) The MFI (mean fluorescence intensity) of SPHK1 and SPHK2. (E) Representative images of TRAP staining of BMM‐derived osteoclasts. Scale bar: 200 μm. (F) Quantification of mature osteoclasts in each group. (G–I) Relative gene expression of *Trap*, *Nfatc‐1*, and *Ctsk*. I: 4T1+PBS; II: 4T1+DOX; III: 4T1+PA; IV: 4T1+PA+DOX; V: 4T1+SKI II; VI: 4T1+DOX+SKI II, VII: 4T1+PA+SKI II, VIII: 4T1+PA+DOX+SKI II. *n* = 3, *p < 0.05, **p < 0.01, ***p < 0.001, and ****p < 0.0001.

To explore whether S1P played a vital role in DOX‐exacerbated osteoclastogenesis described above, we applied SKI II, an SPHK1/2 inhibitor, to block S1P synthesis, before PA and DOX treatment (Figure 5A). Then, CM was collected to induce BMM osteoclastogenesis. As expected, the application of SKI II downregulated the expression of SPHK1/2 and reduced the production of S1P in 4T1 cells (Figure 5B–D and Supporting Information S1: Figure S6A). As a result, the CM from the groups with SKI II pre‐treatment significantly decreased the number of mature osteoclasts in comparison with that of the groups without SKI II pre‐treatment, as indicated by lower reduced TRAP and F‐actin staining (Figure 5E,F and Supporting Information S1: Figure S6B,C). The expression of osteoclast‐related genes, including Trap, Nfatc‐1, and Ctsk, also exhibited a similar trend as histological staining, in which the gene expression declined statistically in the groups with SKI II pre‐treatment (Figure 5G–I). In sum, the results indicate that blocking S1P synthesis inhibits the osteoclastogenesis exacerbated by DOX combined with PA, confirming the regulatory role of S1P in chemotherapy‐caused bone loss.

### 2.6 SPHK1/2 Knockdown in 4T1 Cells Attenuates DOX‐Aggravated Bone Loss in the Obese Mice Bearing Breast Cancer

To further examine the regulatory role of S1P on DOX‐aggravated bone loss, we transfected specific SPHK1 and SPHK2 shRNA lentiviruses (shSPHK1/2) into 4T1 cells. The transfection of shSPHK1/2 successfully downregulated the expression of SPHK1 and SPHK2 in 4T1 cells both on the protein and mRNA levels (Supporting Information S1: Figure S7A–F). The production of S1P in 4T1‐shSPHK1/2 cells was also decreased (Supporting Information S1: Figure S7G). Osteoclast differentiation of BMMs was significantly abated with the CM addition in the presence of SPHK1/2 knockdown (Supporting Information S1: Figure S7H,I).

**FIGURE 6:**
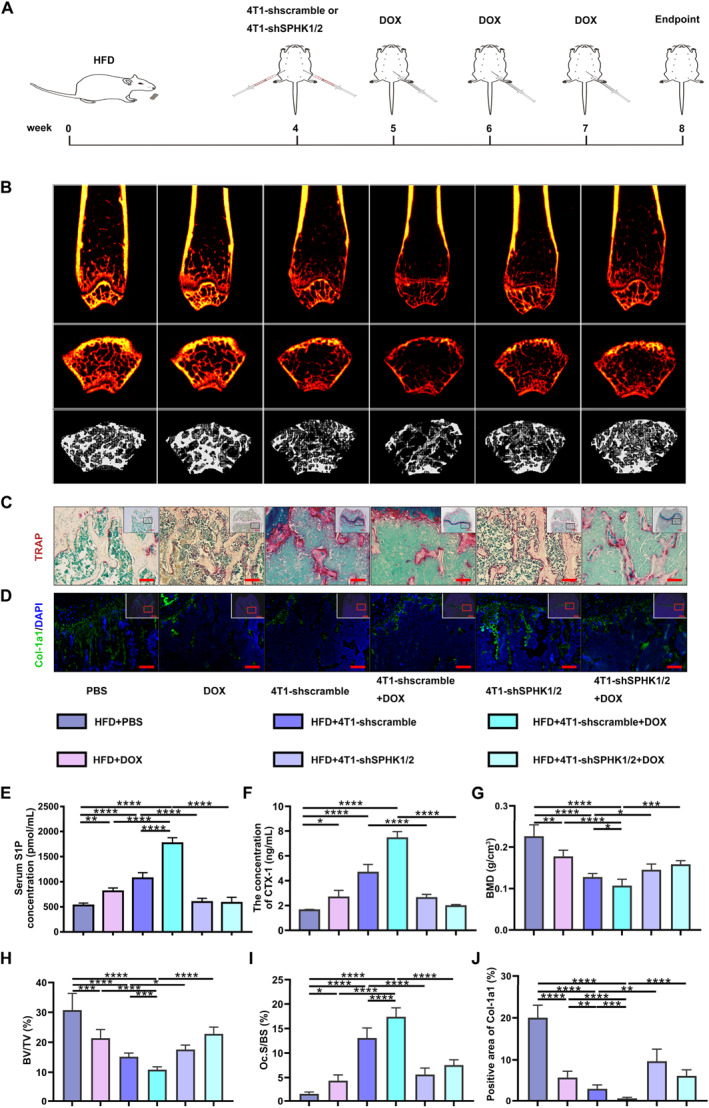
SPHK1/2 knockdown in 4T1 cells attenuates DOX‐aggravated bone loss in the obese mice bearing breast cancer. (A) The schematic diagram of the in vivo experimental protocol. (B) The representative μ‐CT images of the femurs. Upper: coronal sections; Middle: cross sections; Lower: three‐dimensional images. (C) The representative TRAP staining images. (D) The representative images of Col‐1a1 staining. (E, F) The concentrations of S1P and CTX‐I in sera of the mice. (G) BMD of the mice. (H) BV/TV of the mice. (I) The quantification of osteoclasts for TRAP staining. (J) The quantification of Col‐1a1 positive staining. *n* = 4. Scale bar: 100 μm. *p < 0.05, **p < 0.01, ***p < 0.001, and ****p < 0.0001.

The 4T1‐shSPHK1/2 or 4T1‐shscramble cells were then injected into the animal models (Figure 6A). Though significant differences in the body weight of the transfection groups were revealed compared with the control group (HFD+PBS), no statistical change was found among the transfection groups (Supporting Information S1: Figure S8A). The growth of breast cancer was markedly slowed after SPHK1/2 knockdown (Supporting Information S1: Figure S8B). The blood concentration of S1P in the HFD+4T1‐shSPHK1/2+DOX group was remarkably decreased compared with that in the HFD+4T1‐shscramble+DOX group (Figure 6E). Meanwhile, the concentrations of CTX‐I and PINP were both downregulated (Figure 6F and Supporting Information S1: Figure S8C). Notably, the data of H&E staining and μ‐CT scanning displayed that the transfection of shSPHK1/2 into 4T1 cells significantly attenuated DOX‐aggravated bone loss and recovered bone microarchitecture (Figure 6B,G,H and Supporting Information S1: Figure S8D–H).

The quantitative analysis of TRAP and Col‐1a1 staining presented a significant reduction in the osteoclast area (Figure 6C,I) and a substantial increase in the positive area of Col‐1a1 of the HFD+4T1‐shSPHK1/2+DOX group relative to that of the HFD+4T1‐scramble+DOX group (Figure 6D,J). These results indicate that reducing S1P production in 4T1 cells by knocking down SPHK1/2 helps attenuate DOX‐aggravated bone loss in obese mice bearing breast cancer.

### 2.7 FTY720 Alleviates DOX‐Aggravated Bone Loss in the Obese Mice Bearing Breast Cancer

**FIGURE 7:**
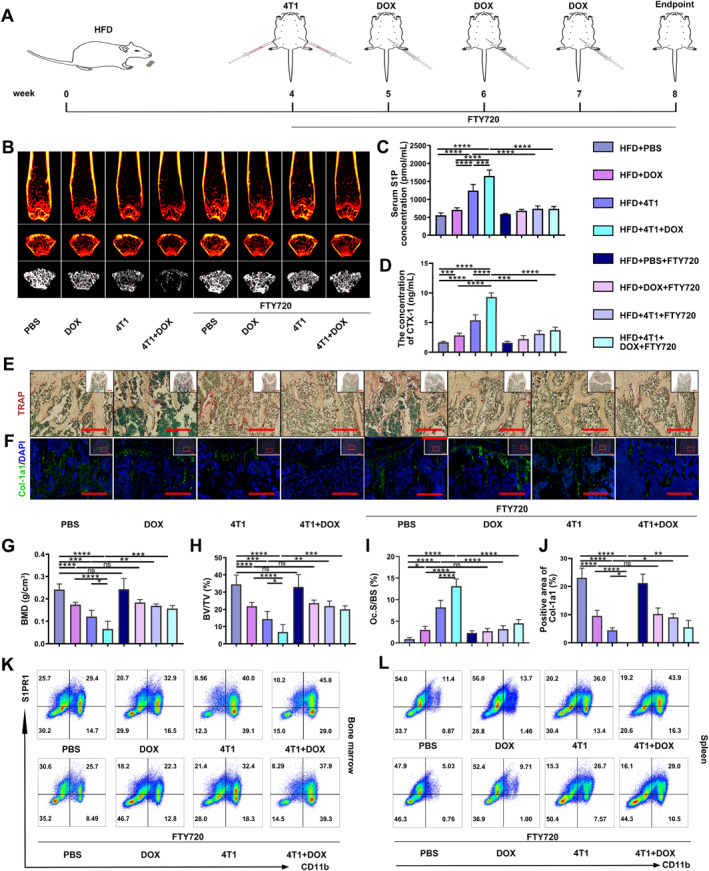
FTY720 attenuates DOX‐aggravated bone loss in the obese mice bearing breast cancer. (A) The schematic diagram of the in vivo experimental protocol. (B) The representative μ‐CT images of the femurs. Upper: coronal sections; Middle: cross sections; Lower: three‐dimensional images. (C) The ELISA detection of serum S1P. (D) The ELISA detection of CTX‐1. (E) The representative TRAP staining images. (F) The representative images of Col‐1a1 staining. (G) BMD. (H) BV/TV. (I) The quantification of osteoclasts for TRAP staining. (J) The quantification of Col‐1a1 positive staining. (K) The analysis of CD11b+S1PR1+ cells in bone marrow by flow cytometry. (L) The analysis of CD11b+S1PR1+ cells in the spleen by flow cytometry. *n* = 4. *p < 0.05, **p < 0.01, ***p < 0.001, and ****p < 0.0001.

FTY720, an S1P antagonist, inhibits the function of S1P by competitively binding to the S1P receptor SPR1 [23]. We treated the mice with daily FTY720 at 1 mg kg^−1^ for 21 days from the beginning of the 4T1 injection (Figure 7A). FTY720 at the administered dose did not induce significant changes in either body weight or tumor volume (Supporting Information S1: Figure S9A,B). Therefore, the potential confounding effects of FTY720‐induced alterations in tumor burden or systemic body weight on the experimental outcomes were excluded. In the obese breast cancer‐bearing mice, the blood concentration of S1P was significantly downregulated after FTY720 application (HFD+4T1+FTY720 vs. HFD+4T1, HFD+4T1+DOX+FTY720 vs. HFD+4T1+DOX, Supporting Information S1: Figure S10B). Meanwhile, FTY720 remarkably reduced the concentrations of CTX‐I and PINP in the tumor‐bearing groups, while no statistical difference was revealed between the non‐tumor groups (Figure 7C,D). The bone phenotype and microarchitecture were also altered assayed by H&E staining and μ‐CT scanning (Figure 7B and Supporting Information S1: Figure S10A). FTY720 significantly attenuated DOX‐aggravated bone loss in cancer‐bearing mice of BMD and BV/TV (Figure 7G,H). Other bone‐related parameters, including BS/BV, BS/TV, Tb. Sp, and Tb. N was also improved (Figure S10E‐H). The positive area of TRAP staining displayed a considerable decline, while the positive area of Col‐1a1 staining was significantly increased after the FTY720 application (Figure 7E,F,I,J). However, no notable differences were revealed between non‐tumor groups. in vivo, FTY720 pretreatment also significantly reduced the proportion of CD11b+S1PR1+ cells in the bone marrow and spleen significantly (Figure 7K,L and Supporting Information S1: Figure S11A,B). Taken together, these results show that FTY720 alleviates DOX‐aggravated bone loss, proving the vital role of S1P in chemotherapy‐induced bone loss in obese mice bearing breast cancer.

## 3 Discussion

Cancer chemotherapy is a critical treatment for patients with malignancies. However, Chemotherapy treatment is associated with various adverse effects, including bone loss. Obesity plays a vital role in the development and spread of breast cancer, and recent studies have indicated that it may also exacerbate cancer‐induced bone loss. However, the underlying mechanism linking chemotherapy and obesity to cancer‐induced bone loss is not yet fully understood. In our study, we identified a mechanism involving the critical factor S1P that connects obesity, chemotherapy, and breast cancer‐induced bone loss. We found that in obese breast cancer‐bearing mice, under the treatment of DOX and fatty acid can lead to an increased concentration of S1P produced by breast cancer cells and increase S1PR1 expression in BMMs. By binding with S1PR1 on the cell surface of osteoclast precursors, the increased S1P promotes osteoclast differentiation through S1PR1/p‐STAT3/NFATc‐1 signal pathway, resulting in the aggravation of DOX‐induced bone loss. Targeting the generation of S1P phosphorylation enzymes SPHK1/2 with SKI II or gene knockdown or S1PR1 with FTY720 significantly rescued DOX‐aggravated bone loss in obese breast cancer‐bearing mice.

Consistent with previous findings [24], we observed that tumors can significantly increase bone loss (Figure 1). In the circumstance of a tumor, a dramatic amount of additional bone loss upon DOX treatment was observed, demonstrating a promoted effect of DOX in cancer cell‐induced bone loss. As reported, emerging evidence underscores the multifaceted effects of DOX on cancer cells, including the production of reactive oxygen species, induction of apoptosis, senescence, autophagy, ferroptosis, and pyroptosis, as well as immunomodulation and DNA damage [25]. Served as a DNA‐damaging agent, DOX has been shown to stimulate SPHK1 expression and activity and promote S1P secretion in a wild‐type p53‐dependent manner [26, 27]. By binding with specific receptors, S1P exported from cancer cells can regulate various cell growth and survival processes [12, 28, 29, 30]. In our study, S1P production increases not only in the 4T1 cells treated by DOX and PA, but also in the blood circulation of obese breast cancer‐bearing mice treated by DOX (Figure 2). There are accumulating reports indicating that S1P is implicated in obesity and breast cancer [18, 31]. Our results demonstrate that obesity can elevate S1P levels. Actually, plasma S1P was found to be elevated in obesity and directly correlated with body mass index and total body fat percentage [32, 33, 34]. Furthermore, in breast‐cancer‐bearing mice with identical levels of obesity, the application of DOX further promoted the generation of S1P, as well as the expressions of SPHK1/2 (Figure 2). The data indicate that S1P is a crucial factor secreted by cancer cells as a result of SPHK1/2 upregulation during DOX‐aggravated bone loss in obese breast cancer‐bearing mice.

Increased blood S1P concentration is a significant factor influencing osteoporotic fractures [13]. In our study, we investigated the impact of DOX on bone remodeling in an S1P‐dependent manner under obesity conditions by adding PA, a fatty acid commonly used to simulate obesity in vitro [35]. The combined administration of DOX and PA significantly enhanced osteoclast differentiation without affecting osteoblast differentiation compared to DOX or PA treatment alone (Figure 3 and Supporting Information S1: Figure S4). Both in vitro and in vivo experiments showed that targeting S1P synthesis using pharmacological SKI II or biological SPHK1/2 shRNAs reversed osteoclastogenesis (Figures 5 and 6 and Supporting Information S1: Figures S6–S8). Taken together, these findings indicate that S1P‐mediated osteoclastogenesis contributes to the aggravated bone loss observed in obese breast cancer‐bearing mice following DOX treatment.

S1P‐S1PR1 signaling, known to modulate osteoclastogenesis by influencing osteoblast RANKL expression, plays a critical role in inflammation and the pre‐metastatic niche in obese breast cancer [36, 37]. Elevated S1P, IL‐6, and p‐STAT3 levels in the pre‐metastatic niche can further drive osteoclastogenesis through the induction of NFATc‐1 expression via the S1PR1 receptor pathway [19, 38, 39, 40]. Our in vitro experiments confirmed that DOX and PA co‐treatment significantly upregulated S1PR1 expression in BMMs exposed to tumor‐conditioned media, as evidenced by increased levels of p‐STAT3, NFATc‐1, and other osteoclast‐related genes and proteins (Figure 3). Although the increase in S1PR1 on the osteoclast precursors (OPCs) surface facilitates the migration of OPCs to sites with elevated S1P concentrations [41], the increased S1PR1 expression of tumor‐induced OPCs under DOX‐assisted obesity conditions in our study may contribute to the altered differentiation of osteoclasts. This is consistent with findings showing that elevated S1P levels trigger inflammatory periodontal resorption via S1PR1 signaling. Pre‐treatment with FTY720, an FDA‐approved inhibitor that suppresses the upregulation of S1PR1, potentially via the ubiquitination pathway [20, 21, 22, 42]. This pharmacological blockade not only reduced CD11b+S1PR1+ cell populations (Figures 4A, 7K,L and Supporting Information S1: Figure S11), but also inhibited osteoclastogenesis via the S1PR1/p‐STAT3/NFATc‐1 pathway (Figures 4, 7, and Supporting Information S1: Figure S10). These results indicate that DOX and PA co‐treatment in breast cancer cells promotes osteoclastogenesis through S1PR1‐mediated signaling.

It has been reported that SKI II acts as a highly selective sphingosine kinase inhibitor with dual effects on SPHK1/2, contributing to anti‐proliferative activity in a variety of cancer cell lines [43]. Pharmacological inhibition with SKI II decreased S1P levels and SPHK1/2 synthesis in breast cancer, and alleviated DOX‐aggravated osteoclastogenesis (Figure 5 and Supporting Information S1: Figure S6). The mechanism was not further studied. We hypothesized that SPHK1/2 is overexpressed in breast cancer cells, indicating a potential therapeutic target. Likewise, biological inhibition with SPHK1/2 shRNAs reduced the blood concentration of S1P and restored DOX‐aggravated bone loss (Figure 6 and Supporting Information S1: Figure S8). The identification and validation with pharmacological and biological interventions further consolidated the vital role of S1P in DOX‐aggravated bone loss in obese breast cancer‐bearing mice.

FTY720, an analog of sphingosine, acts as an antagonist on S1PR1 and prevents the S1P/S1PR1/p‐STAT3/NFATc‐1 amplification. FTY720 administration significantly decreased S1P level and S1P‐mediated osteoclastogenesis, and subsequently mitigated DOX‐aggravated bone loss (Figure 7 and Supporting Information S1: Figure S10). It has been reported that the antagonistic function of FTY720 is induced by the rapid polyubiquitylation, endocytosis, and subsequent proteasomal degradation of S1PR1 [26]. This effect was validated in both our in vivo (Figure 7K,L and Supporting Information S1: Figure S11) and in vitro experiments (Figure 4). The direct impact of FTY720 on sphingolipid metabolism further emphasized the critical role of S1P in DOX‐aggravated bone loss in obese breast cancer‐bearing mice. However, it is essential to rule out the osteoanabolic effects of FTY720 on bone because a previous study has reported that FTY720 administration could protect against ovariectomy‐induced bone loss [44]. Daily treatment with FTY720 at a dose of 1 mg kg^−1^ did not result in any significant change in bone in the control group. Thus, the osteoanabolic effects of FTY720 on bone may be dose‐dependent, which warrants further investigation.

Although our findings demonstrate promising prospects of S1P in the treatment of breast cancer‐induced bone loss, our experiments only focused on the S1P‐mediated crosstalk between the tumor microenvironment and bone microenvironment, and did not fully investigate the systemic conditions of mice following drug administration. Currently in clinical practice, S1PR modulators are primarily used for the treatment of autoimmune diseases such as multiple sclerosis and ulcerative colitis. The adverse reactions monitored are concentrated mainly on the cardiovascular system, liver function, and immune‐related complications. Given these safety concerns, clinical translation of S1P signaling remains challenging. Addressing these limitations will be critical for unlocking the full therapeutic potential of S1P and expanding its applications in fields such as osteoporosis. Thus, it becomes urgent to adopt further strategies to mitigate these limitations in clinical applications.

## 4 Conclusion

In summary, our collective findings identified S1P as a critical molecule connecting obesity, chemotherapy, and breast cancer‐induced bone loss. It is conceivable to speculate that targeting S1P or the involved S1PR1/p‐STAT3/NFATc‐1 axis might provide an experimental basis for future combined chemotherapy‐bone protection strategies.

## 5 Experimental Section

### 5.1 Animals

All protocols involving animals were approved by the Animal Care and Use Committee of Soochow University (202111A0046). Female BALB/c mice, aged 6–8 weeks, were fed with high‐fat diets (HFD) as detailed in Supporting Information S1: Table S1. After 4 weeks, 5 × 10^3^ 4T1 cells were subcutaneously injected into the fourth inguinal mammary glands to establish breast cancer models [45]. One week following the cell injection, the mice received intraperitoneal injections of DOX (5 mg kg^−1^ week^−1^, Sigma, USA) administered weekly for 3 weeks. The HFD‐fed mice were categorized into four groups: control (HFD+PBS), DOX‐treated (HFD+DOX), 4T1‐injected (HFD+4T1), and 4T1‐injected with subsequent DOX treatment (HFD+4T1+DOX).

In some experiments, the SPHK1/2 short hairpin RNA (shSPHK1/2)‐transfected 4T1 cells (4T1‐shSPHK1/2), instead of 4T1 cells, were used to reduce the secretion of S1P in the obese breast cancer‐bearing mice during DOX treatment, namely HFD+4T1+shscramble (nonspecific shRNA control), HFD+4T1+shSPHK1/2, HFD+4T1+DOX+shscramble and HFD+4T1+DOX+shSPHK1/2 group, respectively.

In other experiments, the S1P antagonist FTY720 (1 mg kg^−1^ day^−1^; Selleck, USA) was applied to block the S1P‐S1PR1 signal one week after the 4T1 cell injection during DOX administration.

All the HFD mice were euthanized 4 weeks after the 4T1 cell administration. The samples were harvested for further experiments.

### 5.2 Cell Lines and Cell Culture

Murine 4T1 mammary adenocarcinoma cells, along with MC3T3‐E1 osteoblast precursor cells, were acquired from the Chinese Academy of Sciences. Bone marrow‐derived macrophages (BMMs) were isolated from eight‐week‐old female BALB/c mice and differentiated into osteoclasts using a well‐established protocol [46].

### 5.3 4T1 Cell Treatment

#### 5.3.1 PA and DOX Treatment

4T1 cells were treated with palmitic acid (PA, 20 μΜ, Sigma, USA) for 16 h and then treated with DOX (50 nΜ, Sigma, USA) for another 48 h. According to the treatment, 4T1 cells were divided into 4T1, 4T1+DOX, 4T1+PA, and 4T1+PA+DOX groups, respectively.

#### 5.3.2 SKI II Treatment

In some experiments, 4T1 cells were pre‐treated with SPHK1/SPHK2 antagonist SKI II (10 μΜ, Selleck, USA) for 6–8 h and treated with PA and DOX as above. The CMs were collected after 4T1 cells were resuspended in the plain cell medium for 24 h.

#### 5.3.3 Conditional Medium

To prepare the conditional medium (CM), the 4T1 cells (15 × 10^5^) were resuspended in the plain cell medium. After 24 h, the plain cell medium was harvested and applied as the CM to induce BMMs into osteoclastogenesis or MC3T3‐E1 cell osteogenesis.

### 5.4 Knockdown of SPHK1 and SPHK2 in 4T1 Cells

The expression of SPHK1 and SPHK2 (short as SPHK1/2) in 4T1 cells was knocked down by transfecting with lentiviral‐based shSPHK1/2 or shscramble (nonspecific shRNA control). The sequences of shSPHK1 and shSPHK2 are presented in Supporting Information S1: Table S2. 4T1 cells were transfected with pLVTHM vectors encoding puromycin resistance genes and green fluorescent protein (GFP). The transfected cells were selected over one week using puromycin (15 ng μL^−1^) and G418 (800 μg mL^−1^) and subsequently sorted by flow cytometry. The transfection efficiency was further assessed through quantitative real‐time polymerase chain reaction (qRT‐PCR) and western blot analysis.

### 5.5 Osteoclastogenesis of BMMs

#### 5.5.1 Osteoclast Differentiation

Osteoclast differentiation was conducted following a well‐established method [47]. Briefly, BMMs were subjected to osteoclast differentiation in macrophage colony‐stimulating factor (M‐CSF, 0.03 μg mL^−1^, R&D, USA), receptor activator of nuclear factor‐kappa Β ligand (RANKL, 0.05 μg mL^−1^, R&D, USA), and one‐10th of the CM in a 96‐well plate (2 × 10^4^ cells well^−1^). According to the different CM components, the BMM‐differentiated cells were divided into control, PA, DOX, and PA+DOX groups, respectively.

#### 5.5.2 FTY720 Treatment

In some experiments, BMMs were pre‐treated with FTY720 (0.5 μΜ, Selleck, USA) for 12 h, and then BMMs were subjected to osteoclast differentiation in M‐CSF, RANKL, and one‐fourth, eleventh‐14th of the CM in 24‐well (1 × 10^5^ cells well^−1^) or 96‐well plates (10^4^ cells well^−1^).

#### 5.5.3 RNA and Protein Extraction

Total RNA or protein was extracted from BMM‐differentiated osteoclasts on the third day of osteoclast differentiation for qRT‐PCR or western blot [48, 49]. On the 7th day, Osteoclasts differentiated from BMMs were fixed with 4% paraformaldehyde and stained using a tartrate‐resistant acid phosphatase (TRAP) staining kit (Sigma, USA) following the manufacturer's protocol or incubated with rhodamine‐conjugated phalloidin for cytoskeleton staining using an F‐actin staining kit (Abcam, UK). For quantification of osteoclastogenesis, cells containing three nuclei and above were defined as mature osteoclasts.

### 5.6 Osteoblastogenesis of MC3T3‐E1 Cells

MC3T3‐E1 cells were incubated in the osteogenic medium [50, 51]. Total RNA was extracted on the third day of osteogenic differentiation for further qRT‐PCR analysis. On the seventh day, alkaline phosphatase (ALP) activity was measured using staining and quantification with a BCIP/NBT ALP kit (Beyotime Biotechnology, CN) and an ALP assay kit (Nanjing Jiancheng, CN), respectively. Alizarin Red S staining for mineralized nodules was performed on day 21.

### 5.7 Quantitative Real‐Time PCR (qRT‐PCR)

The extraction and reverse transcription of messenger RNA were performed as described previously [50]. Briefly, total RNA was extracted using TRIzol Reagent (Beyotime Biotechnology, CN), and its concentration was measured using a NanoDrop2000 spectrophotometer (Thermo Fisher Scientific, USA). Complementary DNA was synthesized using a reverse transcription kit (Takara, JPN). qRT‐PCR was performed using SYBR Green Supermix (Bio‐Rad, USA) using the ABI Prism 7900HT system (Bio‐Rad, USA). Gapdh served as the internal control for normalization. Relative gene expression levels were calculated using the comparative Ct (2^−ΔΔCt^) method. Primer sequences of the targeted genes are provided in Supporting Information S1: Table S3.

### 5.8 Western Blot (WB)

Protein lysates were collected and protein concentrations were determined using a BCA Protein Assay Kit (Beyotime Biotechnology, CN). WB was performed on nitrocellulose membranes after incubation with primary antibodies at 4°C overnight, which were as follows: anti‐SPHK1, anti‐SPHK2 (Thermo Fisher Scientific, USA), anti‐S1PR1 (Abcam, USA), anti‐TRAP (Abcam, USA), anti‐NFATc‐1 (anta Cruz Biotechnology, CN), anti‐STAT3 (CST, USA), anti‐p‐STAT3 (CST, USA), and anti‐β‐actin (Abcam, USA). Horseradish peroxidase‐conjugated secondary antibodies (Thermo Fisher Scientific, USA) were incubated for 1 h at room temperature. Protein bands were visualized and semi‐quantified using Image Lab software (Bio‐Rad, USA).

### 5.9 Flow Cytometry Assay (FCA)

Single suspended cells from mouse spleen and tibia bone marrow cavity were isolated [52, 53], and 1 × 106 cells for FCA (BD, USA). After treating BMMs with supernatant or FTY720 for 24 h, 1 × 10^6^ cells were collected and incubated with CD11b‐APC and S1PR1‐FITC antibodies (Thermo Fisher Scientific, USA) for FCA. The FCA data were analyzed with FlowJo software.

### 5.10 Micro‐Computed Tomography (μ‐CT) Scanning and Analysis

Femurs were scanned using Skyscan 1176 (Skyscan, Aartselaar, Belgium) as in our previous study [52]. A region of interest (ROI) was analyzed with CTAn v1.9 software, beginning 0.15 mm proximal to the epiphyseal growth plate and extending 0.4 mm proximally. Bone parameters comprised BMD, bone volume fraction (BV/TV), trabecular thickness (Tb. Th), trabecular number (Tb. N), the ratio of bone volume to bone surface (BS/BV), and trabecular separation (Tb. Sp).

### 5.11 Histology and Immunofluorescent Staining

Mouse femur sections were prepared and stained with hematoxylin and eosin (H&E) for histological analysis [54]. For immunofluorescence analysis, sections were incubated with the primary antibody and secondary goat antibodies to rabbit immunoglobulin G (IgG) (Vector) in turn [55]. Nuclei were stained with DAPI. Images were captured with a Zeiss Axiovert 200 microscope (Zeiss MicroImaging, USA) and analyzed using ImageJ software.

### 5.12 Enzyme‐Linked Immunosorbent Assay (ELISA)

The C‐terminal telopeptide of type I collagen (CTX‐I, a marker for bone resorption, Elabscience, USA) and the amino‐terminal propeptide of type I procollagen (PINP, a marker for bone formation, Elabscience, USA), as well as the concentration of S1P in the blood and bone marrow, were measured with ELISA kits (Echelon Biosciences, USA) following the manufacturer's instructions. Quantification was measured using a microplate reader (Thermo Fisher Scientific, USA) at a wavelength of 450 nm.

### 5.13 Blinding

Mice were randomly assigned to groups by a researcher not involved in data collection or analysis. Researchers performing treatments or surgeries were blinded to group identities. Outcome assessment was conducted by researchers unaware of group assignments.

### 5.14 Statistical Analysis

Statistical analyses were performed using SPSS 13.0 software. Data are expressed as the mean ± standard deviation (SD). Unpaired t‐tests were applied for comparisons between the two groups, while one‐way ANOVA followed by Tukey's post hoc test was used for multiple group comparisons. A *p*‐value of less than 0.05 was considered statistically significant. Sample size estimation was calculated based on the Resource Equation Approach [56].

## Author Contributions

**Yu Zhang:** conceptualization, methodology experiments, validation, writing. **Hao Shen:** conceptualization, methodology experiments, validation, writing. **Junjie Niu:** conceptualization, methodology experiments, validation, writing. **Yingkang Huang:** methodology, investigation. **Can Zhu:** methodology, investigation. **Yi Wang:** methodology, investigation. **Yida Chen:** methodology, validation. **Xinyi Cheng:** methodology, validation. **Huilin Yang:** conceptualization, supervision. **Xianrong Zhang:** conceptualization, supervision. **Hao Chen:** conceptualization, supervision. **Hongbo Zhang:** conceptualization, supervision. **Qin Shi:** conceptualization, supervision, writing – reviewing and editing, and funding acquisition. All authors have read/edited/approved the manuscript.

## Ethics Statement

All protocols involving animals were approved by the Animal Care and Use Committee of Soochow University (202111A0046).

## Conflicts of Interest

Hongbo Zhang is an executive editor for *Smart Medicine* and was not involved in the editorial review or the decision to publish this article. All authors declare that there are no competing interests.

## Supporting information


Supporting Information S1

## Data Availability

The data that support the findings of this study are available from the corresponding author upon reasonable request.
