# Supplementary material for: Targeting Sphingosine‐1‐Phosphate Signaling Attenuates Doxorubicin‐Aggravated Bone Loss in Obese Breast Cancer Mice
Source: Smart Med. 2026 Mar 30;5(2):e70031. doi: 10.1002/smmd.70031 (PMC13131081; doi:10.1002/smmd.70031)
Supplement: Supplementary file 1 — Supporting Information S1 [file SMMD-5-e70031-s001.docx]

**Supporting Information for**

**Targeting Sphingosine-1-Phosphate Signaling Attenuates Doxorubicin-Aggravated Bone Loss in Obese Breast Cancer Mice**

*Yu Zhang, Hao Shen, Junjie Niu, Yingkang Huang, Can Zhu, Yi Wang, Yida Chen, Xinyi Cheng, Huilin Yang, Xianrong Zhang, Hao Chen*, Hongbo Zhang*, Qin Shi**

Y. Zhang, H. Shen, J. Niu, Y. Huang, C. Zhu, Y. Wang, Y. Chen, X. Cheng, H. Yang, Q. Shi

Department of Orthopedics, The First Affiliated Hospital of Soochow University, Orthopedic Institute of Soochow University, 899 Pinghai Road, Suzhou, Jiangsu 215031, China

E-mail: shiqin@suda.edu.cn

Y. Zhang, H. Zhang

Pharmaceutical Sciences Laboratory, Faculty of Science and Engineering, Åbo Akademi University, Turku, 20520, Finland

E-mail: [hongbo.zhang@abo.fi](mailto:hongbo.zhang@abo.fi)

X. Zhang

Department of Orthopedics, Nanfang Hospital, Southern Medical University, 1838 Dadao, Guangzhou, Guangdong, 510515, China

H. Chen

Medical College, Yangzhou University, 136 Jiangyang Road, Yangzhou, Jiangsu 225009, China

E-mail: [hchen329@hotmail.com](mailto:hchen329@hotmail.com)

H. Zhang

Turku Bioscience Centre, University of Turku and Åbo Akademi University, Turku, 20520, Finland

E-mail: hongbo.zhang@abo.fi

Y. Zhang, H. Shen, and J. Niu contributed equally to this work.


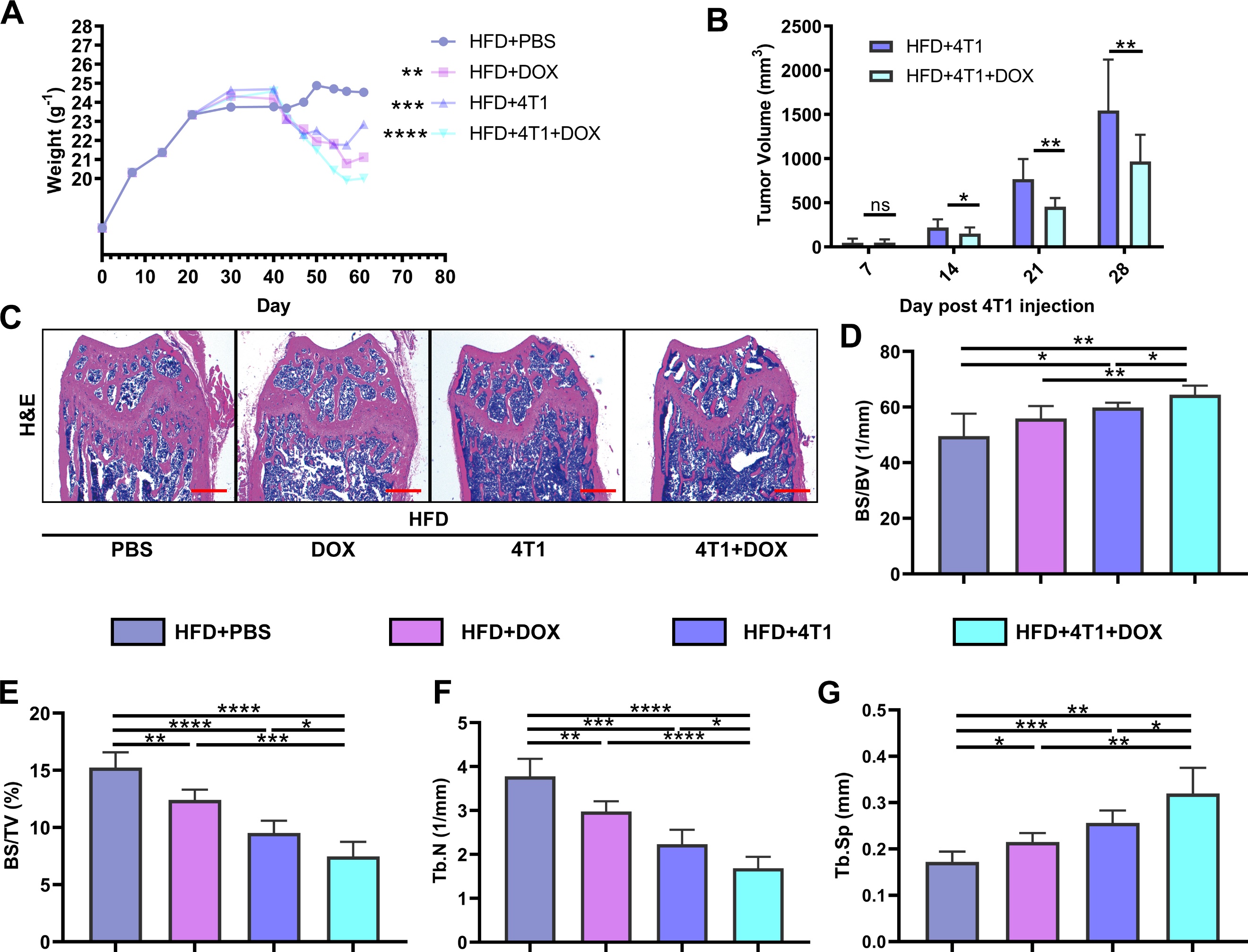


**Figure S1.** DOX treatment exacerbates bone loss in obese mice bearing breast cancer, related to Figure 1. (A) Body weight of the four groups. (B) Tumor volume change. (C) H&E staining images of mouse femurs. (D) BS/BV, (E) BS/TV, (F) Tb. N, and (G) Tb. Sp. n = 4 for each group. Scale bar: 500 μm. *P < 0.05, **P < 0.01, ***P < 0.001, and ****P < 0.0001


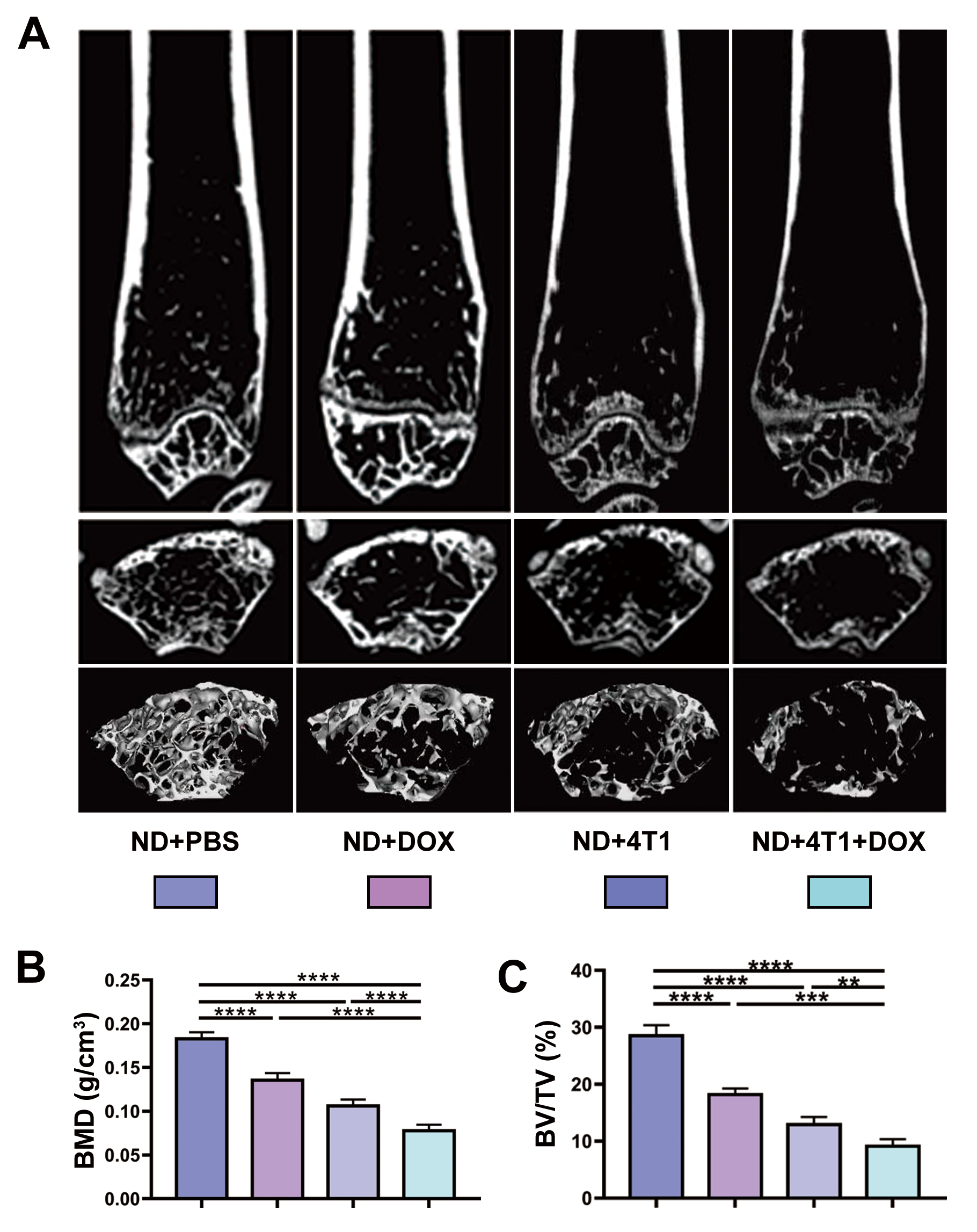


**Figure S2.** DOX exacerbates bone loss in the normal diet mice bearing breast cancer.(A) The representative μ-CT images of the femurs. Upper: coronal sections; Middle: cross sections; Lower: three-dimensional images. (B) BMD and (C) BV/TV of the mice. n=5, **P < 0.01, ***P < 0.001, and ****P < 0.0001


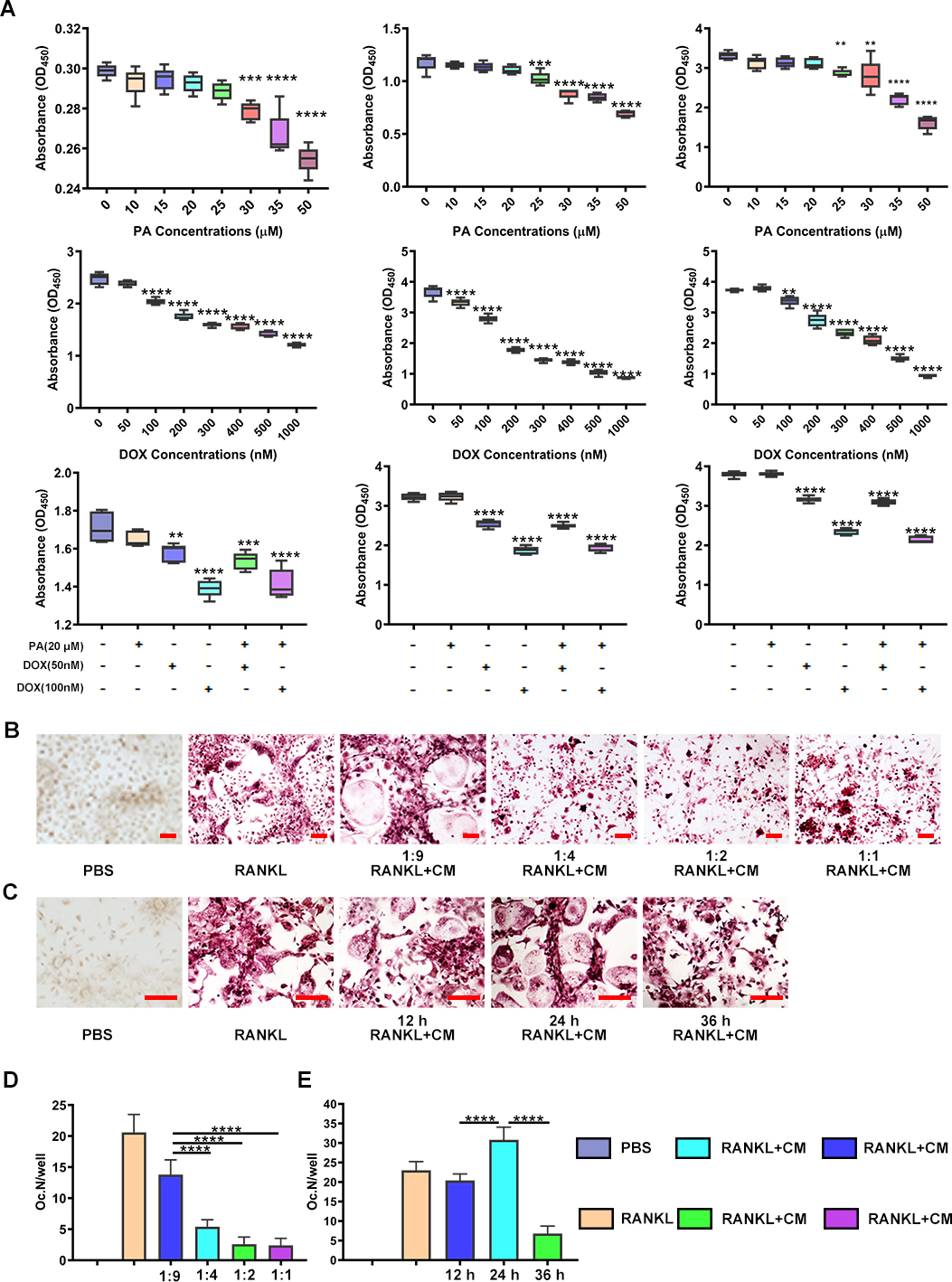


**Figure S3.** The impacts of PA and DOX on cell viability and osteoclast differentiation. (A) Cell viability of 4T1 treated by PA and DOX through CCK-8 assay (n = 5). (B) The representative images of TRAP staining of BMM-derived osteoclasts treated with different ratios of CM to α-MEM. (C) The representative images of TRAP staining of BMM-derived osteoclasts treated at different times. (D) Quantification of osteoclasts using different ratios of CM to α-MEM. (E) Quantification of osteoclasts treated at different times. Scale bar: 200 μm. *P < 0.05, **P < 0.01, ***P < 0.001, and ****P < 0.0001


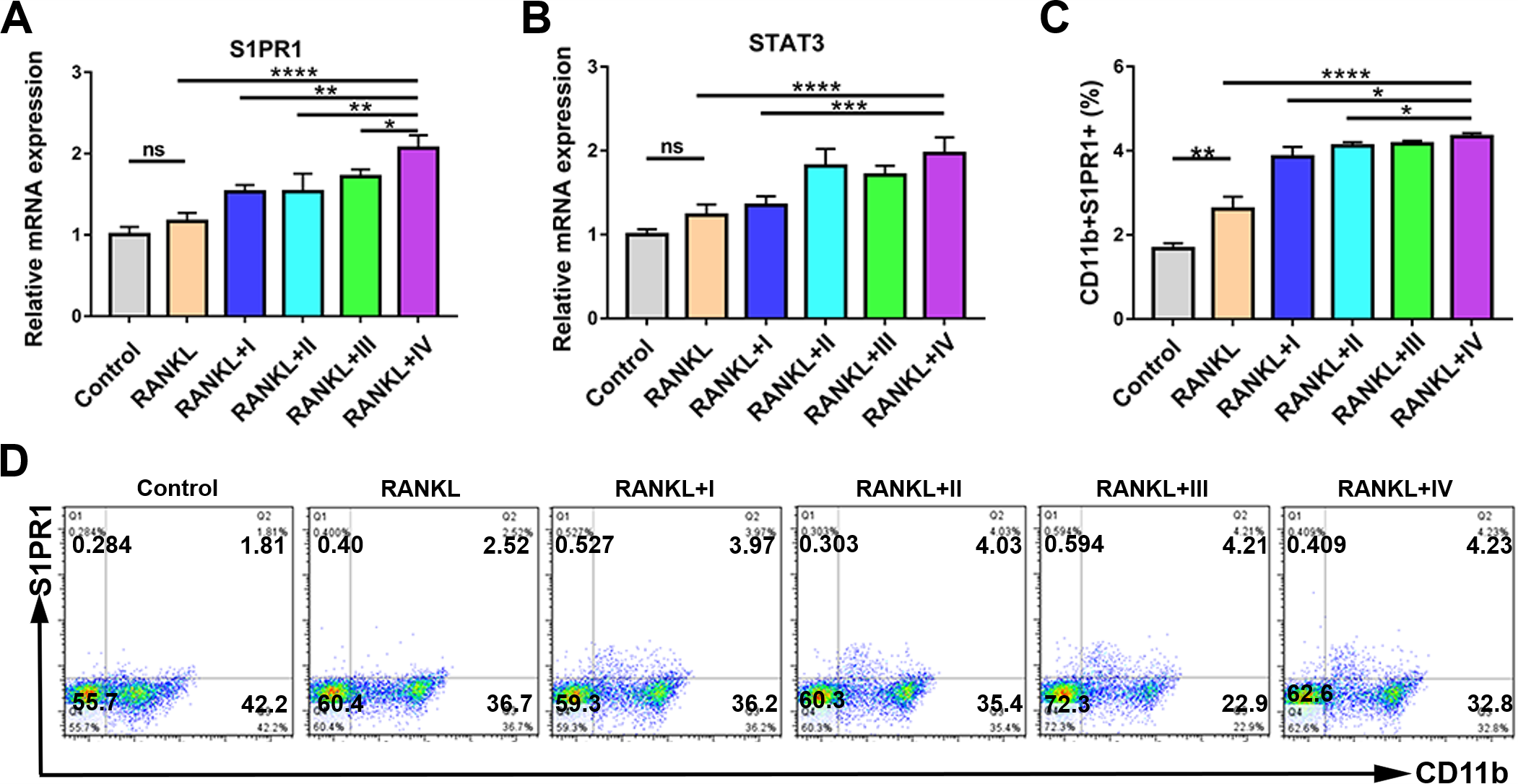


**Figure S4** The effect of supernatant on the S1PR1 expression of BMMs surface. (A) S1PR1 mRNA expression. (B) STAT3 mRNA expression. (C) The proportion statistics of CD11b+S1PR1+ cells (n = 3). (D) Flow cytometry analysis of CD11b+S1PR1+ cells. I: 4T1+PBS; II: 4T1+DOX; III: 4T1+PA; IV: 4T1+PA+DOX. Scale bar: 200 μm. *P < 0.05, **P < 0.01, ***P < 0.001, and ****P < 0.0001


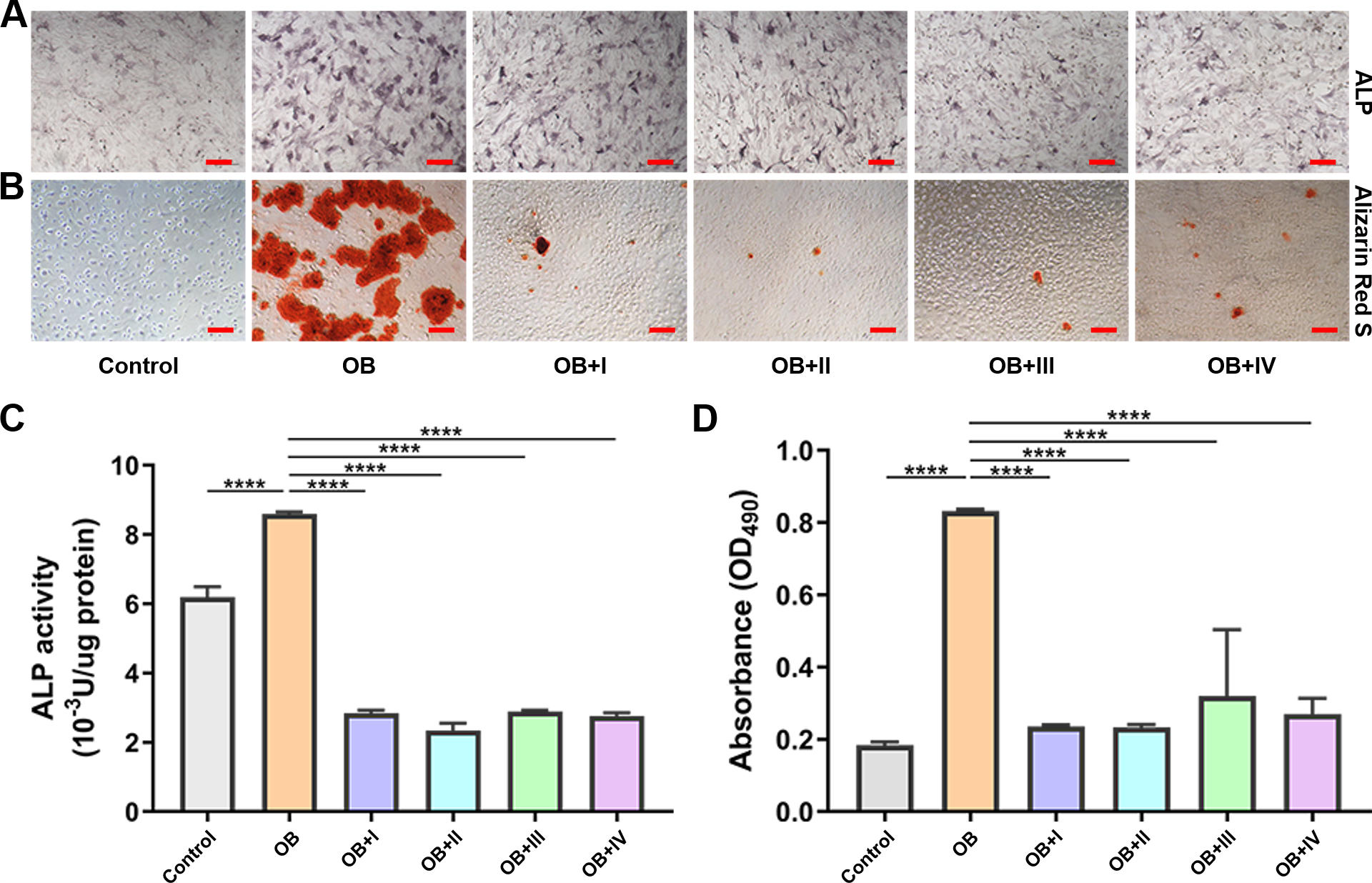


**Figure S5.** The effect of DOX combined with PA on osteoblastogenesis of BMM. (A) Representative images of ALP staining. (B) Representative images of Alizarin Red S staining. (C) Quantification of ALP activity. (D) Quantification of Alizarin Red S staining. I: 4T1+PBS; II: 4T1+DOX; III: 4T1+PA; IV: 4T1+PA+DOX. Scale bar: 200 μm. *P < 0.05, **P < 0.01, ***P < 0.001, and ****P < 0.0001


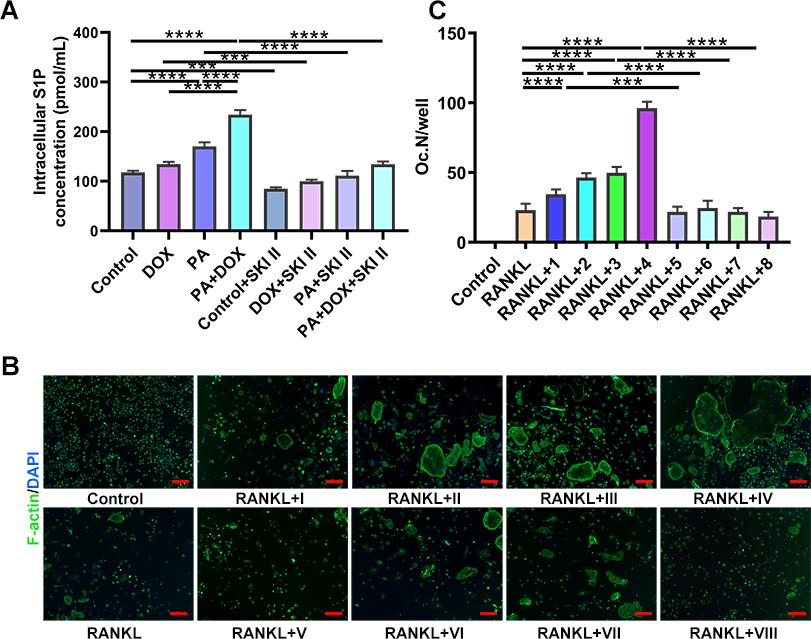


**Figure S6.** SKI II inhibits the osteoclastogenesis exacerbated by DOX combined with PA *in vitro*. (A) The concentration of S1P in tumor cells. (B) Representative images of F-actin staining. Scale bar: 200 μm. (C) Quantification of mature osteoclasts in each group. I: 4T1+PBS; II: 4T1+DOX; III: 4T1+PA; IV: 4T1+PA+DOX; V: 4T1+SKI II; VI: 4T1+DOX+SKI II, VII: 4T1+PA+SKI II, VIII: 4T1+PA+DOX+SKI II. *P < 0.05, **P < 0.01, ***P < 0.001, and ****P < 0.0001


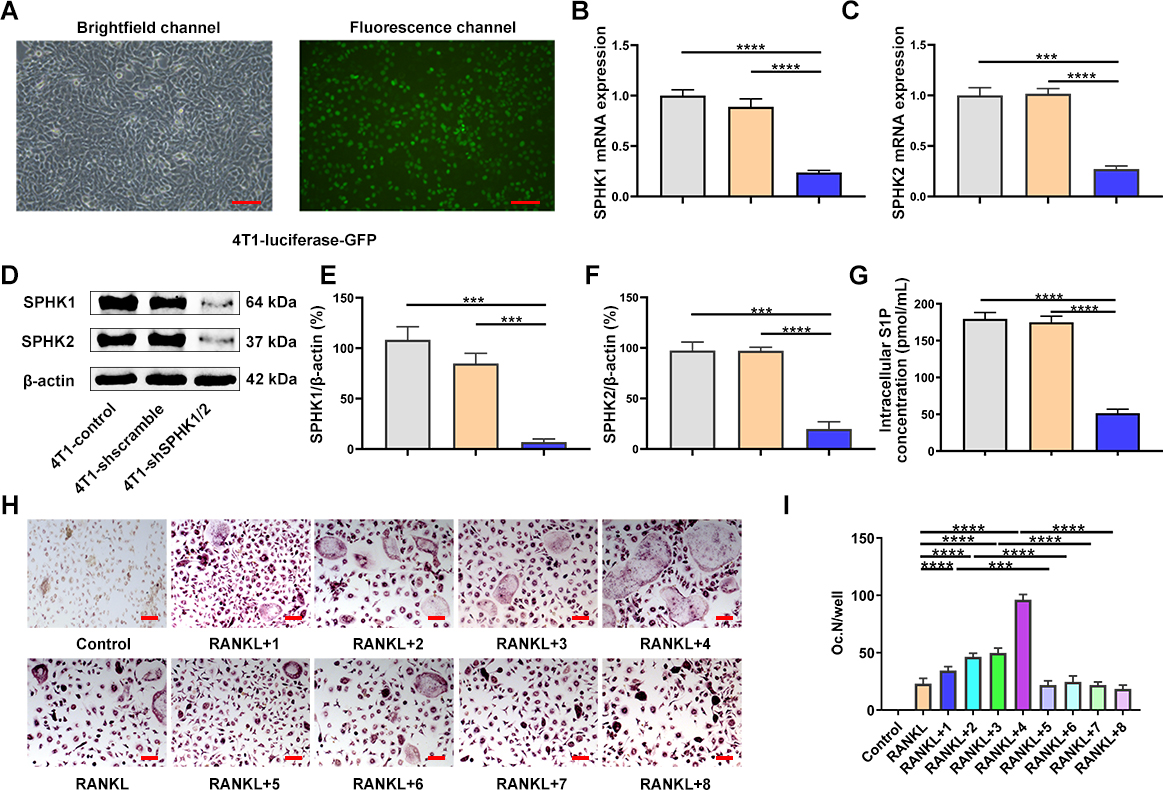


**Figure S7.** The validation of SPHK1/2 knockdown in 4T1 cells. (A) The representative images of 4T1-shSPHK1/2 cells expressing luciferase-GFP. (B) SPHK1 mRNA expression. (C) SPHK2 mRNA expression. (D-F) WB analysis and the quantification of the protein expression of SPHK1 and SPHK2. (G) The S1P concentrations in tumor cells. (H) Representative images of TRAP staining under treatments of different CMs. The CMs were 1: 4T1-shscramble+PBS, 2: 4T1-shscramble+DOX, 3: 4T1-shscramble+PA, 4: 4T1-shscramble+PA+DOX, 5: 4T1-shSPHK1/2+PBS, 6: 4T1-shSPHK1/2+DOX, 7: 4T1-shSPHK1/2+PA and 8: 4T1-shSPHK1/2+PA+DOX. Scale bar: 200 μm. (I) The quantification of mature osteoclasts. Scale bar: 200 μm. *P < 0.05, **P < 0.01, ***P < 0.001, and ****P < 0.0001.


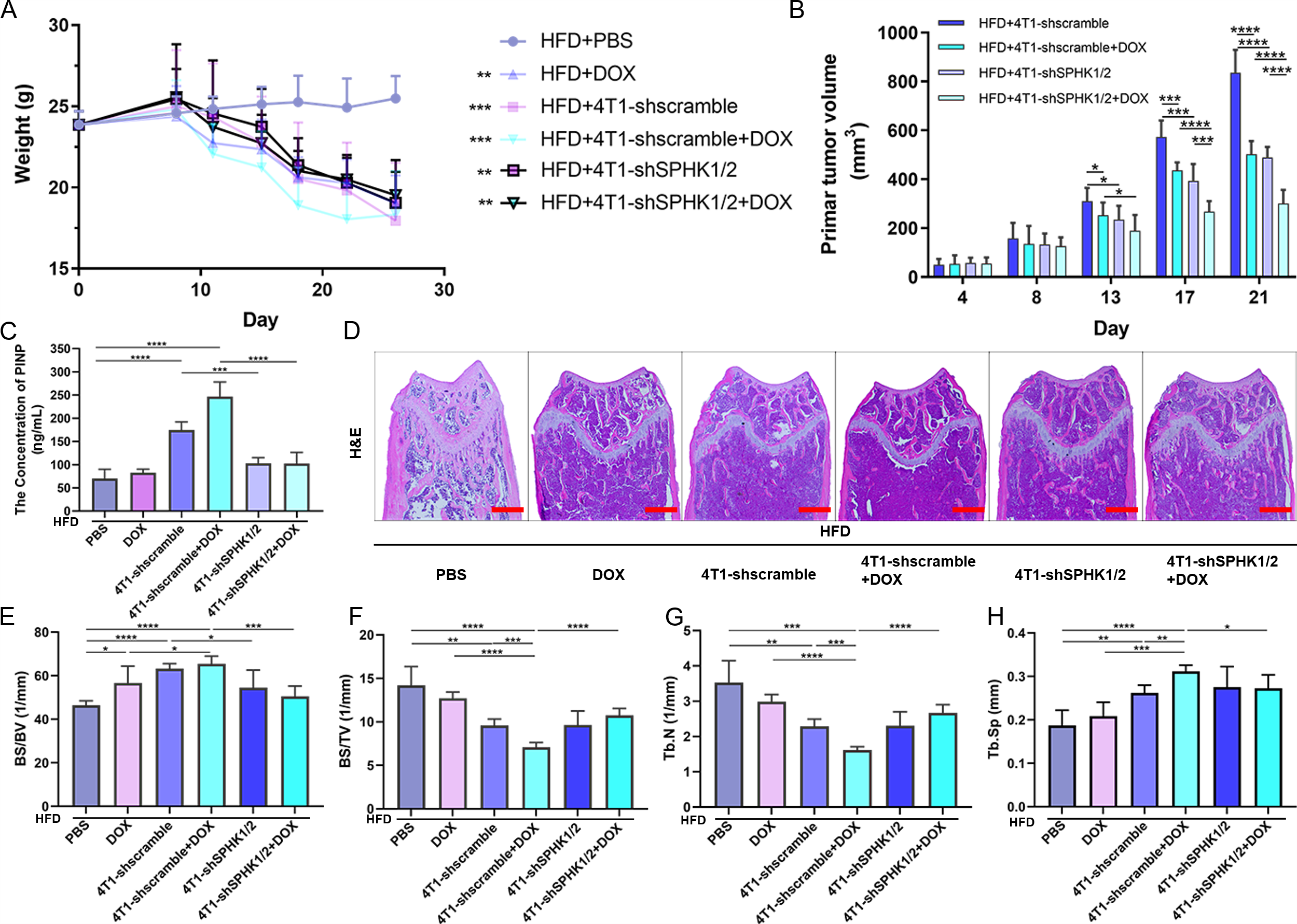


**Figure S8.** SPHK1/2 knockdown in 4T1 cells attenuates DOX-aggravated bone loss in obese mice bearing breast cancer, related to Figure 6. (A) Body weight of the mice in different groups. (B) Tumor volume change. (C) Blood concentration of PINP. (D) H&E staining images of mouse femurs. (E-H) Parameters of bone microarchitecture, including BS/BV, BS/TV, Tb. N, and Tb. Sp. n = 4. Scale bar: 500 μm. *P < 0.05, **P < 0.01, ***P < 0.001, and ****P < 0.0001.


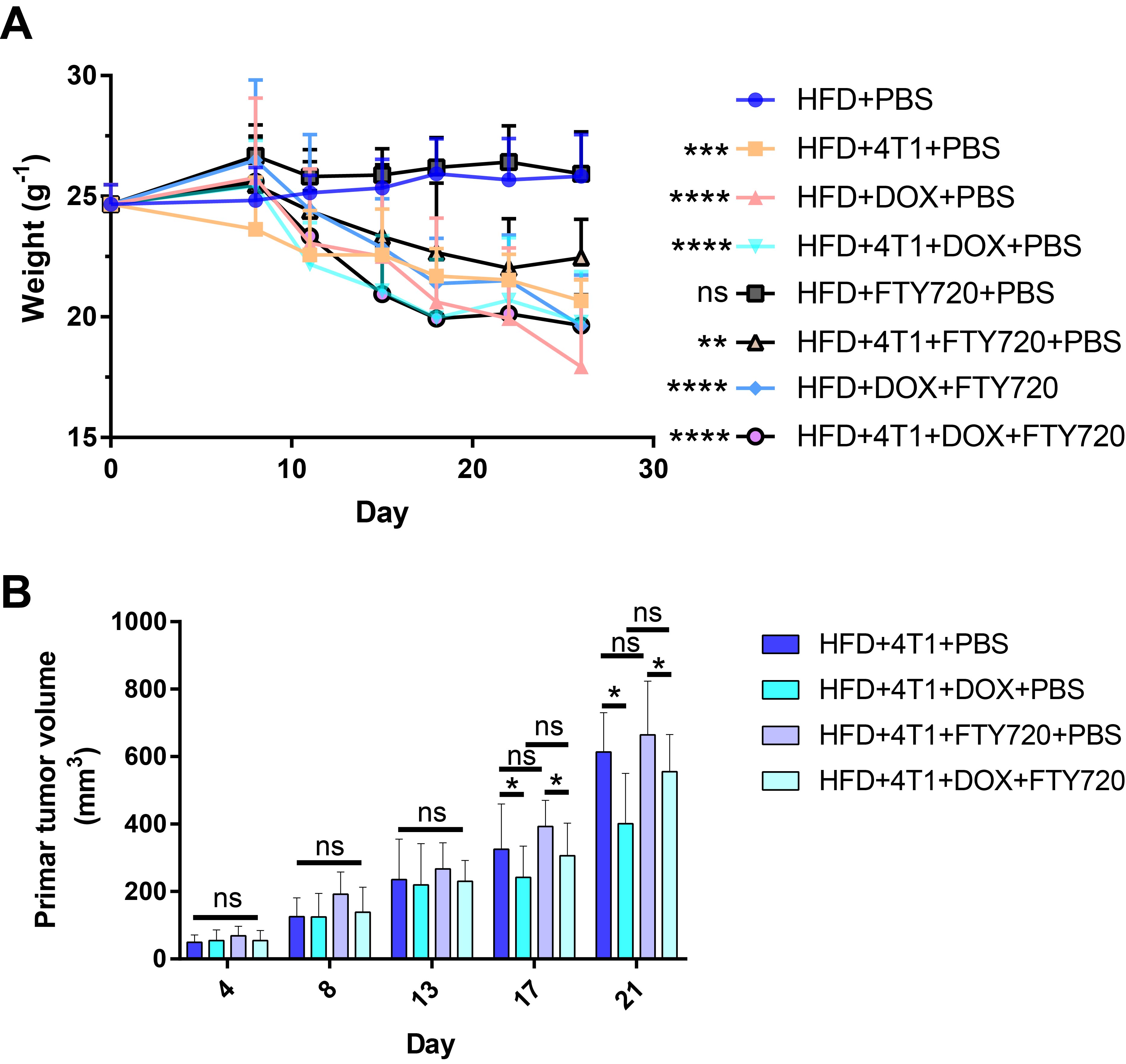


**Figure S9.** FTY720 attenuates DOX-aggravated bone loss in obese mice bearing breast cancer, related to Figure 7. (A) Body weight of the mice in different groups. (B) Tumor volume change. n = 5, *P < 0.05, **P < 0.01, ***P < 0.001, and ****P < 0.0001


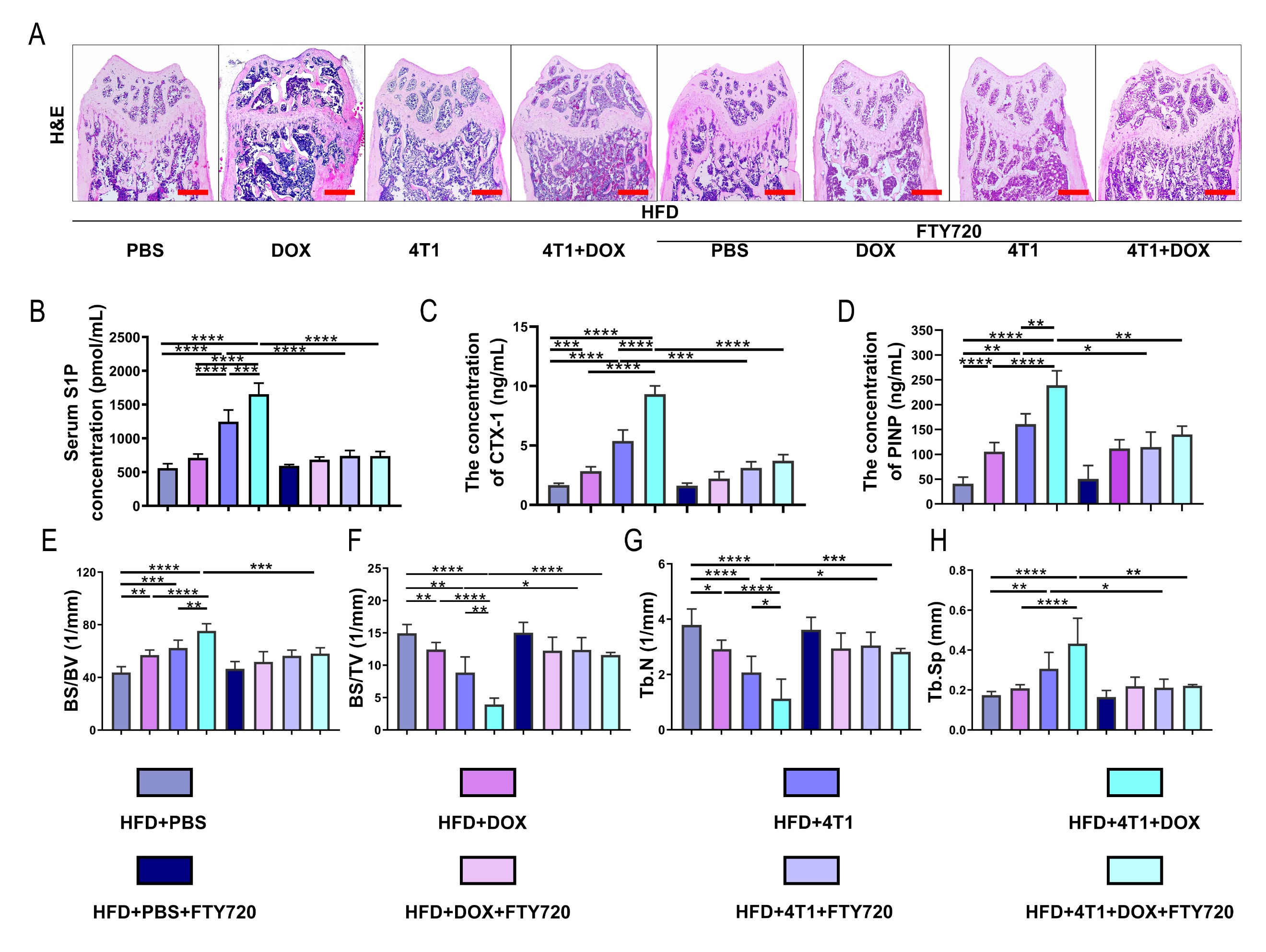


**Figure S10.** FTY720 attenuates DOX-aggravated bone loss in obese mice bearing breast cancer, related to Figure 7. (A) H&E staining images of mouse femurs. (B) The blood concentration of S1P. (C) The blood concentration of CTX-1. (D) The blood concentration of PINP. (E-H) Parameters of bone microarchitecture, including BS/BV, BS/TV, Tb. N, and Tb. Sp. n = 4. Scale bar: 500 μm. *P < 0.05, **P < 0.01, ***P < 0.001, and ****P < 0.0001


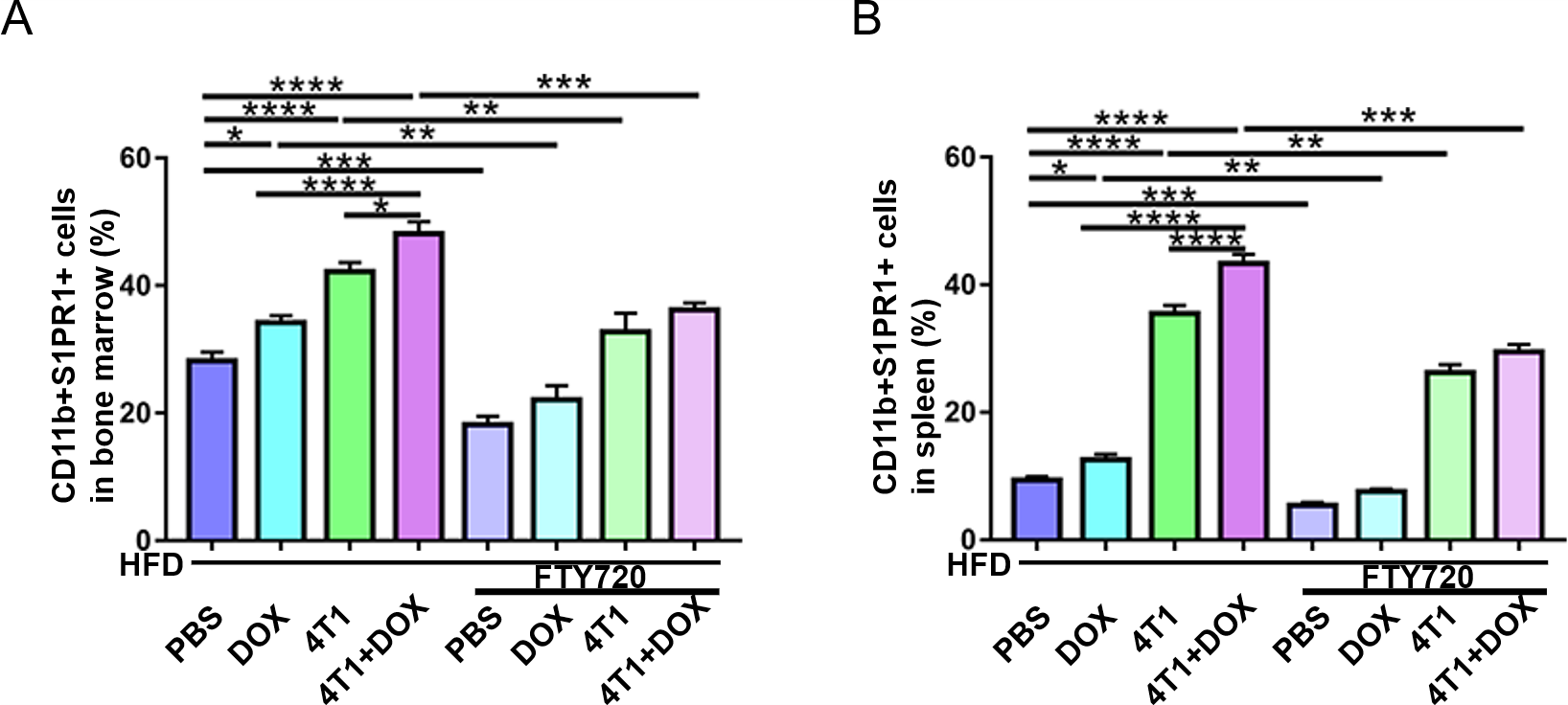


**Figure S11.** The proportion statistics of CD11b+S1PR1+ cells. (A) The proportion statistics of CD11b+S1PR1+ cells in bone marrow, (n = 3). (B) The proportion statistics of CD11b+S1PR1+ cells in the spleen, (n = 3). *P < 0.05, **P < 0.01, ***P < 0.001, and ****P < 0.0001

**Table S1. The formula of the high-fat diet, related to Figure 6, and Figure 7.**

| Compositions | Percentage（%） |
| --- | --- |
| Barley flour | 22.0 |
| Lard | 20.0 |
| Soy flour | 18.5 |
| Corn flour | 14.0 |
| Cholesterol | 1.0 |
| Bran | 7.5 |
| Yeast | 1.0 |
| Fish meal | 7.5 |
| Bone meal | 1.5 |
| Egg yolk powder | 5.0 |
| Inorganic salts | 1.0 |
| Sodium Cholate | 1.0 |

**Table S2. The sequences of shSPHK1 and shSPHK2, Related to Figure 6.**

| shRNA | Sequence (5’-3’) |
| --- | --- |
| sh*Sphk1* | GGTACGAGCAGGTGACTAATG |
| sh*Sphk2* | AACCTCATACAGACAGAACGA |

**Table S3. The primer sequences of qRT-PCR, Related to Figure 2, Figure 3, Figure 5, and Figure 6.**

| Genes | Primer sequence（5’-3’） |
| --- | --- |
| *Stat3* | Forward GTCTGCAGAGTTCAAGCACCT |
|  | Reverse TCCTCAGTCACGAT CAAGGAG |
| *S1pr1* | Forward ATGGTGTCCACTAGCATCCC |
|  | Reverse CGATGTTCAACTTGCCTGTGTAG |
| *Ctsk* | Forward ATGTGGGTGTTCAAGTTTC |
|  | Reverse TCAATGCCTCCGTTCT |
| *Dc-stamp* | Forward TACGTGGAGAGAAGCAAGGAA |
|  | Reverse ACACTGAGACGTGGTTTAGGAAT |
| *Nfatc-1* | Forward TGGGAGATGGAAGCAAAGAC |
|  | Reverse ATAGAAACTGACTTGGACGGG |
| *Trap* | Forward AGACCCAATGCCACCC |
|  | Reverse GGACCTCCAAGTTCTTATC |
| *Runx2* | Forward GACACTGCCACCTCTGACTT |
|  | Reverse GATGAAATGCTTGGGAACTG |
| *Opn* | Forward CTTTCACTCCAATCGTCCCTAC |
|  | Reverse CAGAAACCTGGAAACTCCTAGAC |
| *Ocn* | Forward CGCTCTGTCTCTCTGACCTC |
|  | Reverse CACTACCTTATTGCCCTCCTG |
| *Sphk1* | Forward TCCTGGAGGAGGCAGAGATA |
|  | Reverse GCTACACAGGGGTTTCTGGA |
| *Sphk2* | Forward AAATCACCCCTGAATTGCTG |
|  | Reverse ATGCCTTCCCACTCACTCAG |
| *Gapdh* | Forward GGTGAAGGTCGGTGTGAACG |
|  | Reverse CTCGCTCCTGGAAGATGGTG |
